# Supplementary material for: A scalable microarrayed organoid platform for precision modeling of intestinal epithelial damage and recovery
Source: Cell Rep Methods. 2026 Aug 14;6(10):101551. doi: 10.1016/j.crmeth.2026.101551 (PMC13721746; doi:10.1016/j.crmeth.2026.101551)
Supplement: Supplementary file 1 [file mmc1.pdf]

Cell Reports Methods, Volume *6*

Supplemental information

A scalable microarrayed organoid platform for precision modeling of intestinal epithelial damage and recovery

Marine Meyer, Aline Roch, Juliane Dervaux, Laurent Ferrier, Evarist Planet, Julien Duc, Camilla Ceroni, Mariana Costa, Brice Chambrin, Jens Stolte, Sylviane Métairon, Abhilash Kannan, Sylke Hoehnel-Ka, Marine R.-C. Kraus, Patrick Descombes, Nathalie Brandenberg, Mario Noti, and Omid Mashinchian

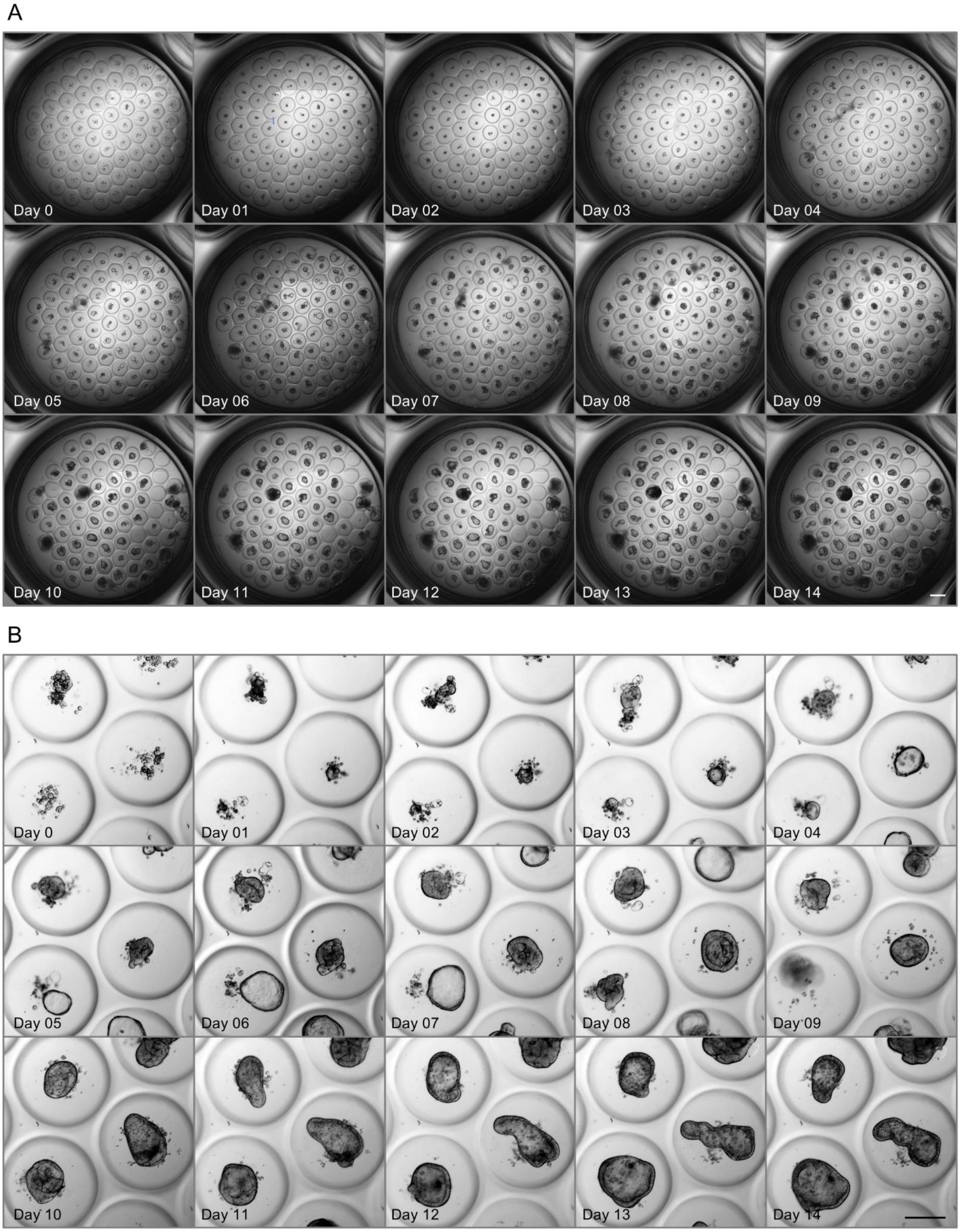

Figure S1. Time-Lapse Microscopy of Intestinal Organoid Growth and Development over 14 Days, related to Figure 1. A. Representative low-magnification brightfield images capturing the overall growth and expansion pattern of multiple organoids cultured within microwells, tracked daily from Day 0 to Day 14. The scale bar denotes consistent magnification across all images: 500 μm. **B.** High-magnification brightfield images illustrating morphological changes and detailed structural development of individual intestinal organoids within microwells over the same 14-day period, highlighting the transition from initial small cellular clusters to mature, differentiated organoid structures exhibiting distinct epithelial morphologies and lumen formation. The scale bar indicates uniform magnification for all high-magnification panels: 300 μm.

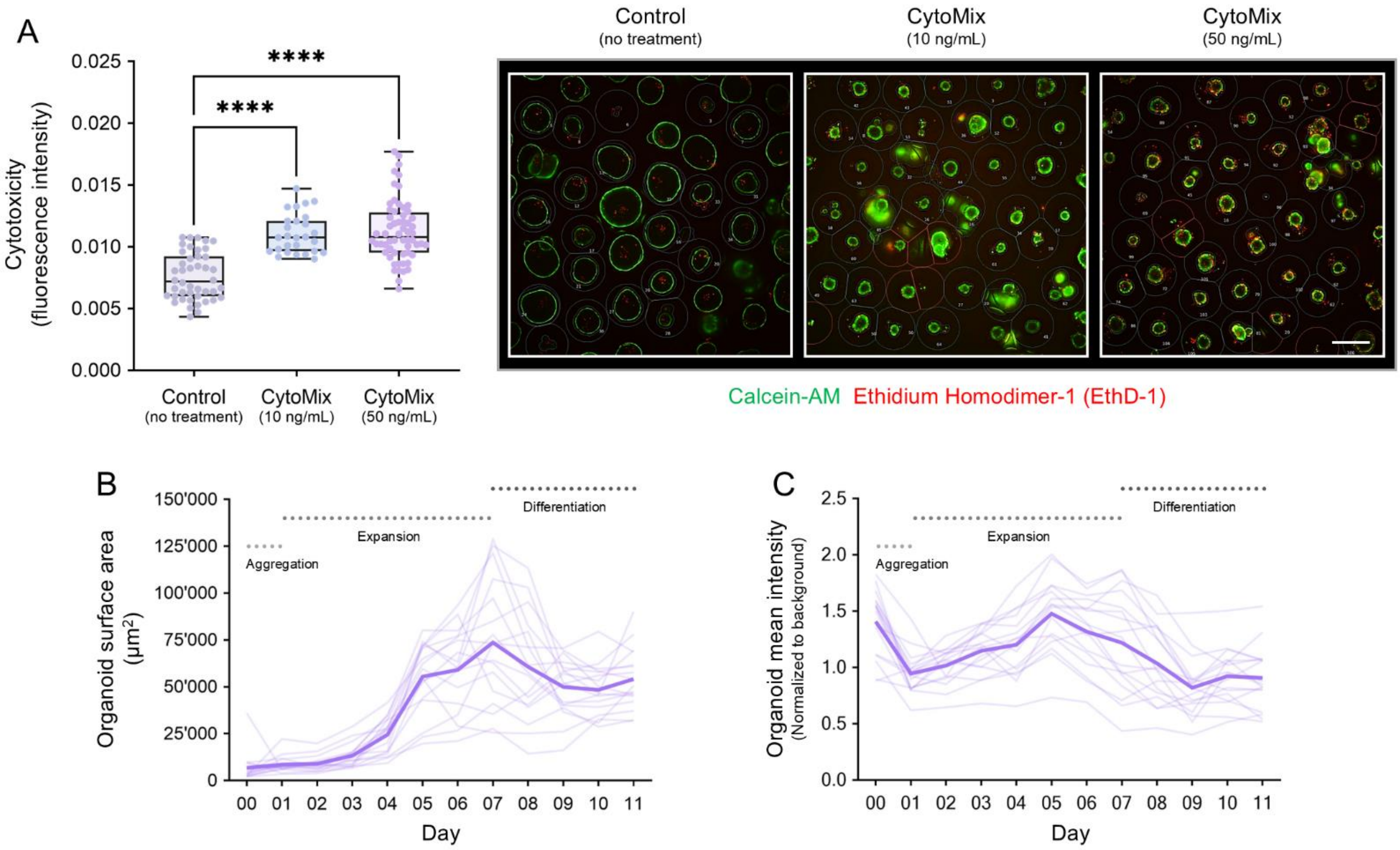

Figure S2. Cytotoxicity and Morphodynamic Assessment of Intestinal Organoids During Development and Cytokine-Induced Stress, related to Figures 1 and 2. A. Cytotoxicity was assessed by measuring the fluorescence intensity of Ethidium Homodimer-1 (EthD-1) in control (untreated), CytoMix-treated (10 ng/mL), and high-dose CytoMix-treated (50 ng/mL) organoids after 48 hours. Quantitative data are presented as box plots, with statistical significance indicated by asterisks ($n \geq 27$ organoids). Each dot represents an individual organoid. Two-way ANOVA with Bonferroni's post-test was performed using GraphPad Prism (****$p < 0.0001$). Representative fluorescence microscopy images depict viable cells (green; Calcein-AM) and compromised or dead cells (red; Ethidium Homodimer-1 (EthD-1)) under each treatment condition. Images are shown as maximum intensity projections across 15 z-planes, with a step size of 20 µm. Scale bars represent 500 µm and indicate uniform magnification across all images. **B.** Quantitative analysis of organoid surface area dynamics during aggregation, expansion, and differentiation phases. Individual organoid trajectories are shown in light purple, and the mean trajectory is highlighted in dark purple (mean, $n \geq 17$ organoids). **C.** Corresponding organoid mean intensity measurements normalized to background intensity, reflecting changes in organoid density and structural complexity during culture progression (mean, $n \geq 17$ organoids).

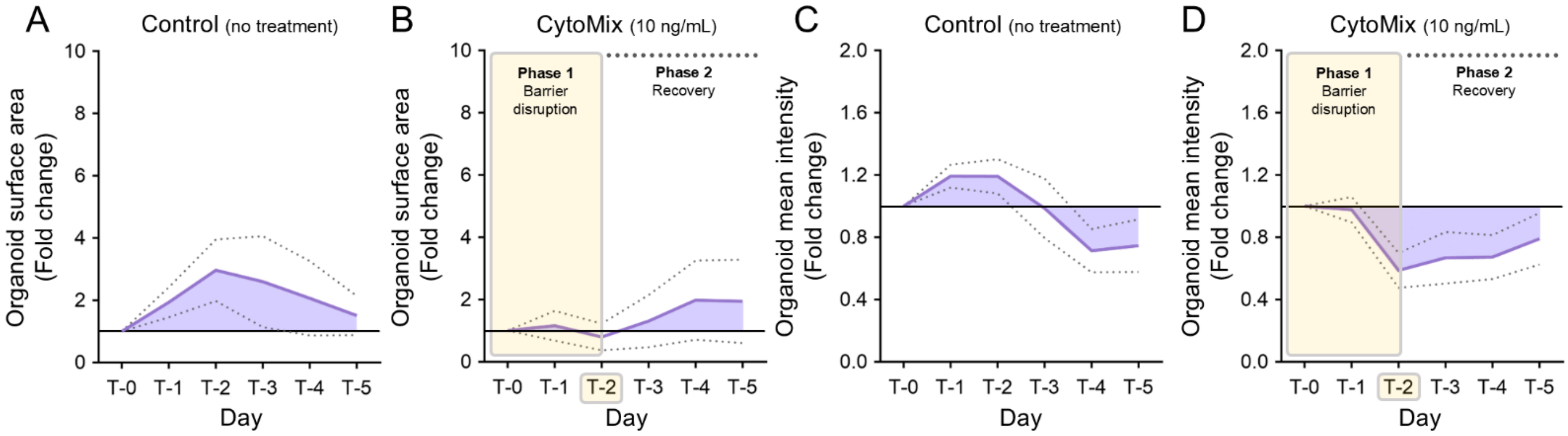

Figure S3. Longitudinal feature dynamics underlying IORS calculation in untreated and CytoMix-challenged intestinal organoids, related to Figure 3. A–B. Time-course analysis of organoid surface area, expressed as fold change relative to baseline, in untreated control organoids and organoids exposed to CytoMix (10 ng/mL). **C–D**. Time-course analysis of normalized organoid mean intensity, expressed as fold change relative to baseline, in untreated control organoids and CytoMix-treated organoids. The shaded region indicates Phase 1, corresponding to cytokine-induced epithelial barrier disruption, while the subsequent period represents Phase 2, corresponding to post-cytokine recovery. Solid lines represent mean values, shaded purple bands indicate variability around the mean, and dotted lines indicate the spread of individual organoid trajectories. These longitudinal feature dynamics provide the underlying input parameters used for calculating the Intestinal Organoid Recovery Score (IORS) and illustrate the reversible epithelial injury phenotype induced by CytoMix 10 ng/mL.

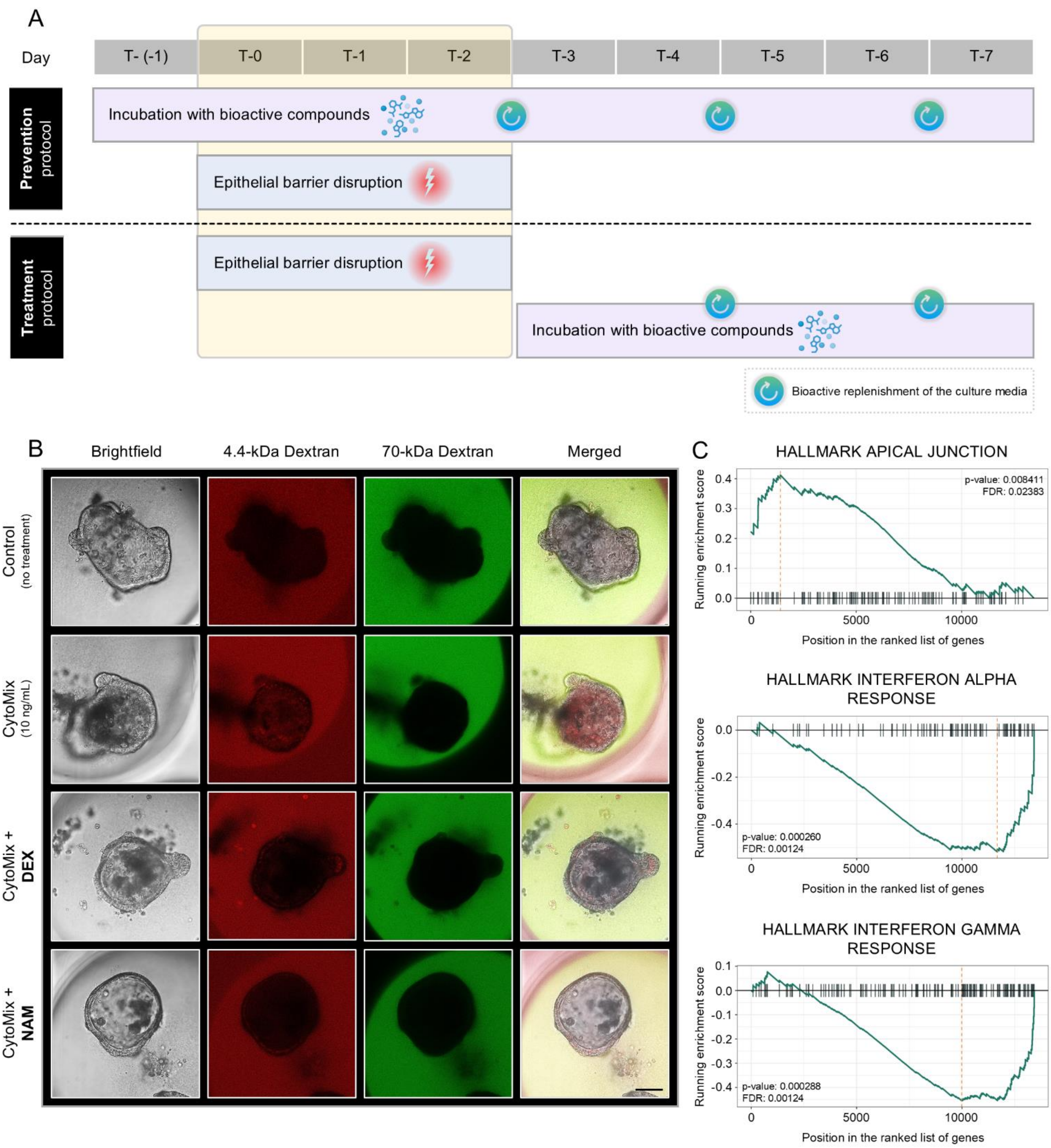

Figure S4. Preventive and Therapeutic Effects of Dexamethasone (DEX) and Nicotinamide (NAM) on Cytokine-Induced Barrier Disruption Assessed by Permeability Assays and Transcriptomic Profiling, related to Figures 4 and 5. A. Schematic outline of experimental timelines and treatment protocols: Prevention (Therapeutic agents administered prior to cytokine exposure) and treatment (interventions initiated post-epithelial disruption) protocols, specifying the timing of cytokine exposure, recovery phases, and administration of bioactive compounds. **B.** Representative microscopy images showcasing barrier integrity using fluorescently labeled dextran permeability assays (4.4-kDa and 70-kDa dextrans) under various experimental conditions: Control (no treatment), CytoMix (10 ng/mL) alone, CytoMix plus dexamethasone (DEX), and CytoMix plus nicotinamide (NAM). Scale bars reflect uniform magnification across all images: 50 µm. **C.** Gene set enrichment analysis (GSEA) plots demonstrate significantly altered hallmark gene signatures related to epithelial barrier function (apical junction integrity) and inflammatory signaling pathways (Interferon Alpha and Interferon Gamma Responses), highlighting significant modulation following DEX treatment. Enrichment scores, nominal *p*-values, and False Discovery Rates (FDR) values are provided.
