# Supplementary material for: A scalable microarrayed organoid platform for precision modeling of intestinal epithelial damage and recovery
Source: Cell Rep Methods. 2026 Aug 14;6(10):101551. doi: 10.1016/j.crmeth.2026.101551 (PMC13721746; doi:10.1016/j.crmeth.2026.101551)
Supplement: Supplementary file 2 [file mmc2.pdf]

Cell Reports
Methods

Article

A scalable microarrayed organoid platform for precision modeling of intestinal epithelial damage and recovery

Graphical abstract

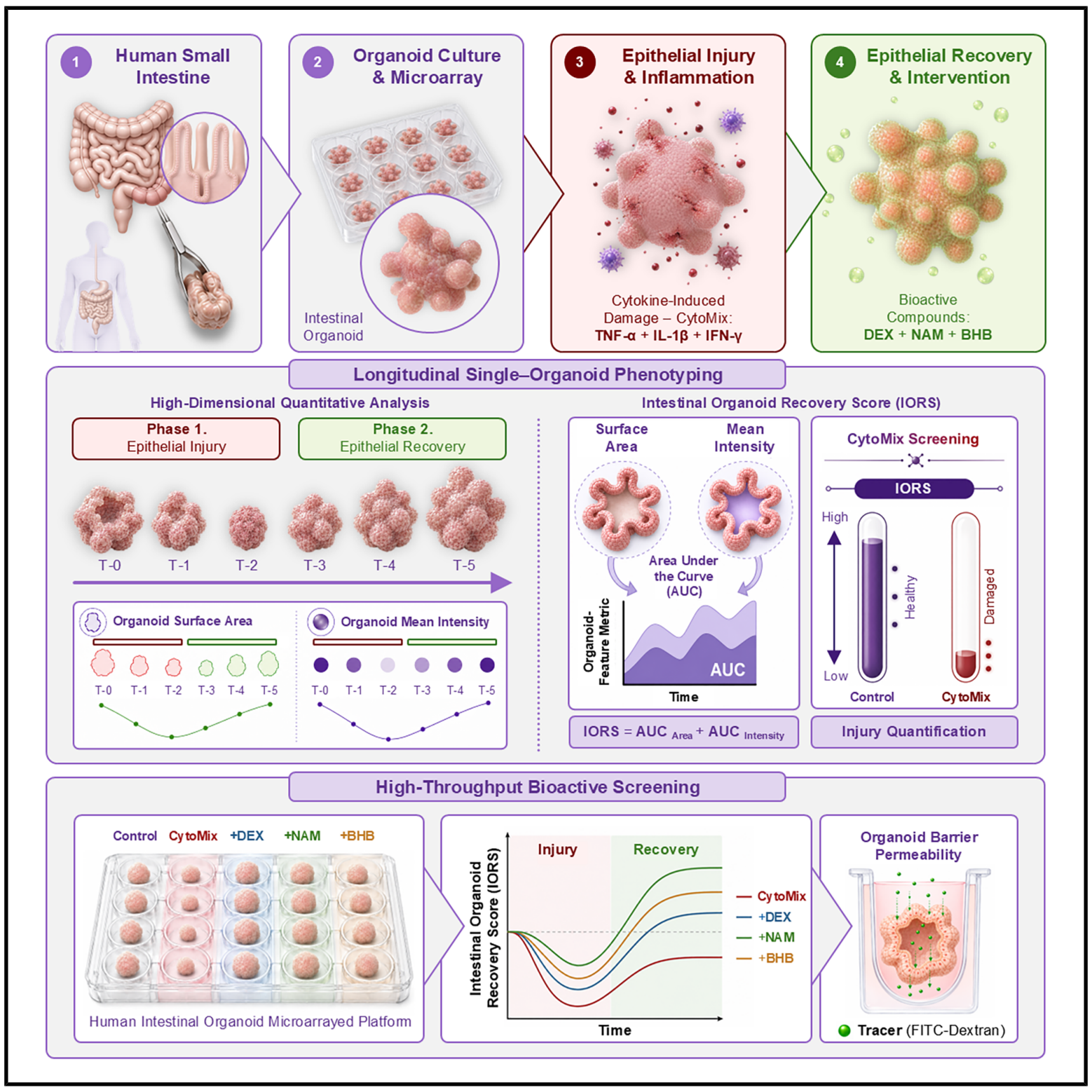

Highlights

- Scalable microarray enables single-organoid inflammatory phenotyping
- IORS quantifies dynamic epithelial damage-recovery trajectories
- CytoMix-induced epithelial programs overlap with Crohn's disease signatures
- DEX and NAM improve barrier recovery through distinct pathways

Authors

Marine Meyer, Aline Roch, Juliane Dervaux, ..., Nathalie Brandenberg, Mario Noti, Omid Mashinchian

Correspondence

nathalie.brandenberg@epfl.ch (N.B.), mario.noti@rd.nestle.com (M.N.), omid.mashinchian@rd.nestle.com (O.M.)

In brief

Meyer et al. present a microarrayed human intestinal organoid platform for longitudinal, single-organoid analysis of cytokine-induced epithelial injury and recovery. The study introduces IORS to quantify epithelial restitution and applies the platform to benchmark bioactives that support barrier recovery under controlled inflammatory stress.

Meyer et al., 2026, Cell Reports Methods 6, 101551
October 19, 2026 © 2026 The Authors. Published by Elsevier Inc.
https://doi.org/10.1016/j.crmeth.2026.101551

CellPress

Cell Reports Methods

CellPress
OPEN ACCESS

Article

A scalable microarrayed organoid platform for precision modeling of intestinal epithelial damage and recovery

Marine Meyer,[1,7] Aline Roch,[1,7] Juliane Dervaux,[1,7] Laurent Ferrier,[2,7] Evarist Planet,[3,8] Julien Duc,[3,8] Camilla Ceroni,[1] Mariana Costa,[2] Brice Chambrin,[2] Jens Stolte,[4] Sylviane Métairon,[4] Abhilash Kannan,[5] Sylke Hoehnel-Ka,[1] Marine R.-C. Kraus,[2] Patrick Descombes,[4] Nathalie Brandenberg,[1,9,*] Mario Noti,[2,9,*] and Omid Mashinchian[2,6,9,10,*]

[1]DOPPL S.A., EPFL Innovation Park, 1015 Lausanne, Switzerland
[2]Nestlé Institute of Health Sciences, Nestlé Research, Société des Produits Nestlé S.A., 1015 Lausanne, Switzerland
[3]Nexco Analytics, EPFL Innovation Park, 1015 Lausanne, Switzerland
[4]Nestlé Institute of Food Safety and Analytical Sciences, Nestlé Research, Société des Produits Nestlé S.A., 1000 Lausanne, Switzerland
[5]Scientific IT and Data Office, Nestlé Research, Société des Produits Nestlé S.A., 1000 Lausanne, Switzerland
[6]Nestlé Product Technology Center, Société des Produits Nestlé S.A., 3510 Konolfingen, Switzerland
[7]These authors contributed equally
[8]These authors contributed equally
[9]Senior author
[10]Lead contact
*Correspondence: nathalie.brandenberg@epfl.ch (N.B.), mario.noti@rd.nestle.com (M.N.), omid.mashinchian@rd.nestle.com (O.M.)
https://doi.org/10.1016/j.crmeth.2026.101551

MOTIVATION Dynamic epithelial injury and repair are central to intestinal inflammatory diseases, but scalable human models that quantify these processes over time remain limited. We developed a microarrayed human intestinal organoid platform to enable longitudinal, single-organoid-resolution analysis of cytokine-induced epithelial disruption and recovery. By combining machine learning-based image analysis, transcriptomics, permeability assays, and the intestinal organoid recovery score, this method provides a standardized framework for phenotype-based screening and mechanistic assessment of epithelial barrier restitution under controlled inflammatory stress.

SUMMARY

Chronic inflammatory bowel diseases (IBDs), including Crohn's disease, are characterized by relapsing-remitting intestinal inflammation associated with epithelial barrier dysfunction. Existing preclinical models of IBD have limited capacity to quantitatively capture dynamic epithelial damage and recovery, which hinders the translation of research findings into new therapeutic approaches. Here, we establish a scalable, microarrayed 3D human intestinal organoid platform enabling high-throughput, single-organoid-resolution phenotyping of cytokine-induced epithelial injury and recovery. By integrating time-resolved morphological imaging, transcriptomics, permeability assays, and the intestinal organoid recovery score (IORS), we quantify injury-recovery trajectories and identify responder organoids following preventive or therapeutic intervention. CytoMix-challenged organoids display epithelial inflammatory programs with transcriptional overlap to Crohn's disease biopsy signatures. Dexamethasone, nicotinamide, and β-hydroxybutyrate improved epithelial recovery, with dexamethasone and nicotinamide modulating distinct inflammatory and barrier-associated pathways. Collectively, this single-organoid-resolution approach establishes a standardized platform for modeling epithelial inflammatory damage and recovery, enabling phenotype-driven precision medicine applications in gastrointestinal disease.

INTRODUCTION

Chronic inflammatory bowel diseases (IBDs), such as Crohn's disease (CD)[1] and ulcerative colitis (UC),[2] represent a growing global health burden characterized by episodic and progressive mucosal inflammation, often culminating in irreversible damage to the intestinal epithelial barrier.[3–7] This specialized barrier plays a pivotal role in maintaining homeostasis by regulating nutrient absorption,

This is an open access article under the CC BY license (http://creativecommons.org/licenses/by/4.0/).

immune surveillance, and host-microbiota interactions.[8,9] Disruption of this barrier is increasingly recognized not only as a hallmark but also as a driver of disease progression and therapeutic resistance.[6,9] While considerable progress has been made in understanding the immunological underpinnings of IBD,[10,11] the dynamic processes governing epithelial barrier dysfunction and its regeneration remain poorly resolved. This gap largely stems from the limitations of existing preclinical models, which often fail to recapitulate the complex, temporal interplay of inflammation-induced epithelial injury and recovery observed in human disease.[12–15] Conventional two-dimensional (2D) cell monolayers and static transwell cultures, while widely used, do not adequately mimic the three-dimensional (3D) architecture or functional heterogeneity of the intestinal epithelium.[16–18] Furthermore, *in vivo* models often lack scalability and human-specific physiological relevance, limiting their translational utility in drug development and personalized medicine.[13,15] Recent advances in 3D organoid technologies[19,20] have begun to bridge this gap by providing *ex vivo* platforms derived from patient tissues that retain key features of their tissue of origin and are capable of partially preserving or recapitulating the patient-specific inflammatory signature.[21–24] However, the lack of high-throughput, quantitative, and standardized methodologies to monitor and interrogate single-organoid dynamics has hampered their widespread adoption for precision modeling of intestinal inflammation and regenerative biology. To address these challenges, we developed a robust, scalable, and microarrayed 3D human intestinal organoid platform that enables high-throughput, single-organoid resolution phenotyping of inflammation-induced epithelial barrier dysfunction and recovery. By integrating longitudinal imaging, quantitative morphological metrics, transcriptomics, and functional assays, our platform captures the dynamic trajectory of individual organoids as they respond to defined inflammatory challenges and therapeutic interventions. The core of this approach lies in our introduction of the ‘‘intestinal organoid recovery score (IORS)’’, a composite, time-resolved metric that integrates organoid size and intensity to quantitatively differentiate responder phenotypes during inflammatory challenge and subsequent recovery. Using this platform, we established a cytokine-driven inflammatory model (CytoMix) that induces epithelial damage phenotypes relevant to gut barrier disruption in CD. The resulting transcriptomic responses in organoids showed overlap with key inflammatory pathways enriched in mucosal biopsies from CD patients,[25] underscoring the translational relevance of the model. Notably, the regenerative capacity of organoids was preserved and quantifiable, enabling the screening of bioactives with healing potential. We demonstrated the efficacy of preventive interventions using dexamethasone (DEX), nicotinamide (NAM), and β-hydroxybutyrate (BHB), which differentially modulated inflammation and promoted epithelial regeneration via distinct molecular mechanisms. Importantly, transcriptomic profiling revealed that DEX primarily suppressed pro-inflammatory signaling pathways, while NAM activated epithelial barrier maintenance and metabolic homeostasis programs, including apical junction integrity[26] and Hedgehog signaling.[27,28] These divergent yet complementary mechanisms highlight the potential for combinatorial therapeutic strategies targeting both inflammation and epithelial resilience. Functional validation using dextran permeability assays confirmed the barrier-protective effects of these treatments, further supporting their therapeutic potential. Collectively, our work establishes a next-generation organoid-based screening platform ensuring scalability and reproducibility while capturing the phenotypic and transcriptional complexity of epithelial responses to inflammation. By enabling high-throughput, quantitative, and mechanistic dissection of epithelial barrier dynamics at single-organoid resolution, this approach provides a foundation for standardized epithelial phenotyping and future patient-specific applications in gastrointestinal inflammatory diseases. It offers a transformative framework for candidate interventions, evaluating patient-specific responses, and uncovering regenerative mechanisms underlying mucosal healing in IBD.

RESULTS

Development and characterization of a scalable, high-throughput 3D human intestinal organoid microarrayed platform

We have bioengineered a scalable, microarrayed, 3D human intestinal organoid platform, specifically optimized for high-throughput and precision modeling of intestinal inflammation and epithelial regeneration at single-organoid resolution (Figure 1A). This advanced platform employs individual microcavity arrays,[29] enabling reproducible and quantitative evaluation of organoid growth dynamics with high precision and consistency. Bright-field microscopy provided detailed insights into the morphological progression of organoids cultured within microcavities at various stages of their development, including initial aggregation on day 0, proliferative expansion on days 4 and 7, and early differentiation on days 9 and 11 (Figure 1B; Figure S1A). High-magnification imaging further illustrated detailed morphological features, demonstrating reproducible organoid formation and maturation within the microcavity platform (Figure 1B; Figure S1B). On day 8, additional organoid analyses were performed using confocal microscopy. This involved staining for DNA (blue), epithelial polarization (F-actin, green), cell proliferation (Ki-67, magenta), and epithelial integrity (E-cadherin, magenta) (Figure 1C). Immunofluorescence imaging highlighted apical-in epithelial polarization within organoids, with proliferative cells predominantly localized to budding and growth-associated regions, alongside clear epithelial barrier formation indicated by E-cadherin expression (Figure 1C). Together, our advanced microarrayed intestinal organoid platform enables precise monitoring of organoid growth dynamics, demonstrating consistent morphological progression through aggregation, expansion, and differentiation stages, along with clear spatial distribution of proliferative regions and epithelial integrity as confirmed by immunofluorescence microscopy. These data highlight the platform's robustness for probing epithelial responses to inflammatory and other exogenous stressors at single-organoid resolution and for evaluating therapeutic interventions.

Decoding cytokine-induced epithelial disruption and recovery in intestinal organoids through multimodal imaging and high-dimensional feature analysis

Systematic screening of a broad panel of cytokines is critical for establishing a physiologically relevant inflammatory organoid

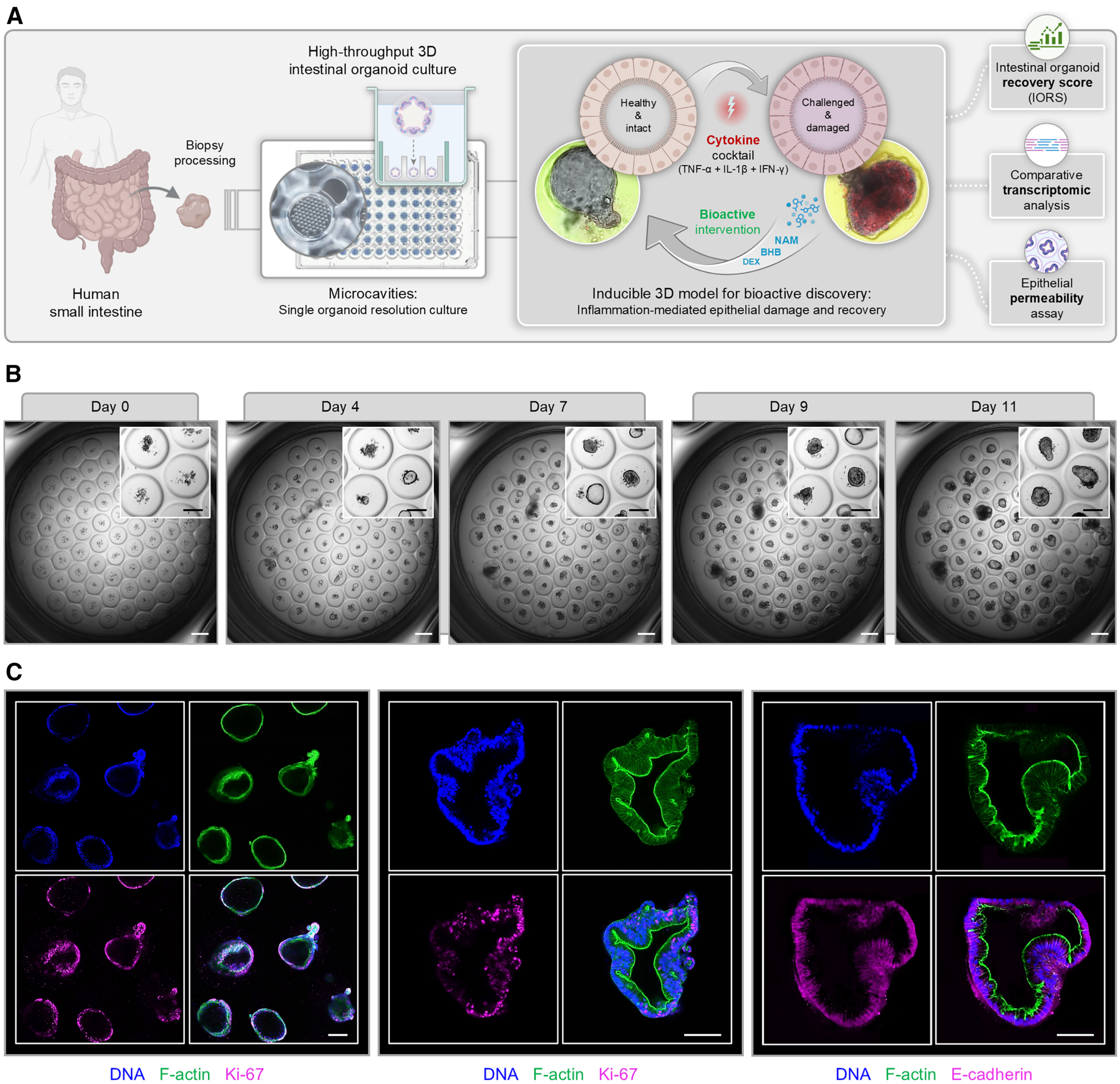

Figure 1. A microarrayed high-throughput 3D intestinal organoid culture platform for *in vitro* modeling of inflammation-induced epithelial dysfunction, regeneration dynamics, and bioactive compound screening

(A) Schematic illustrating the establishment of a high-throughput 3D intestinal organoid culture derived from human small intestine biopsies. Organoids are individually cultured in microcavity arrays to enable single-organoid resolution. This setup allows for the assessment of transcriptional, structural (intestinal organoid recovery score, IORS), and functional (intestinal permeability) changes in response to inflammation-induced barrier dysfunction and recovery, and thus, efficacy testing of bioactives.

(B) Bright-field microscopy images demonstrate distinct morphological stages of organoid development within microcavities in steady-state conditions, including aggregation (day 0), expansion (days 4 and 7), and differentiation (days 9 and 11). Insets show high-magnification views of organoid morphological features at each stage. Scale bars: 500 μm for microcavities and 300 μm for insets.

(C) Confocal microscopy images of representative organoids on day 8 stained for DNA (blue), F-actin (green), Ki-67 (magenta, proliferation marker), and E-cadherin (magenta, epithelial integrity marker). Scale bars represent 200 μm for the organoid array and 100 μm for the magnified single organoid images.

See also Figure S1.

model, as different cytokines can elicit distinct cellular responses and inflammatory pathways.[30,31] This approach enables the identification of optimal cytokine combinations and concentrations that mimic specific inflammatory conditions, thereby enabling controlled disruption of the intestinal epithelial barrier and accurately modeling the dynamic processes of epithelial dysfunction and repair *in vitro*.[32] CytoMix, a pro-inflammatory cytokine cocktail composed of tumor necrosis factor-alpha (TNF-α), interleukin-1 beta (IL-1β), and interferon-gamma (IFN-γ), is widely applied to *in vitro* gut models to simulate inflammatory conditions and assess epithelial barrier dysfunction.[33] We first assessed the impact of cytokine-induced inflammation on intestinal epithelial integrity under different CytoMix[33] dosing regimens in comparison with IFN-γ[34] alone (Figure 2A). Morphological alterations were monitored via brightfield microscopy at sequential time points during two distinct experimental

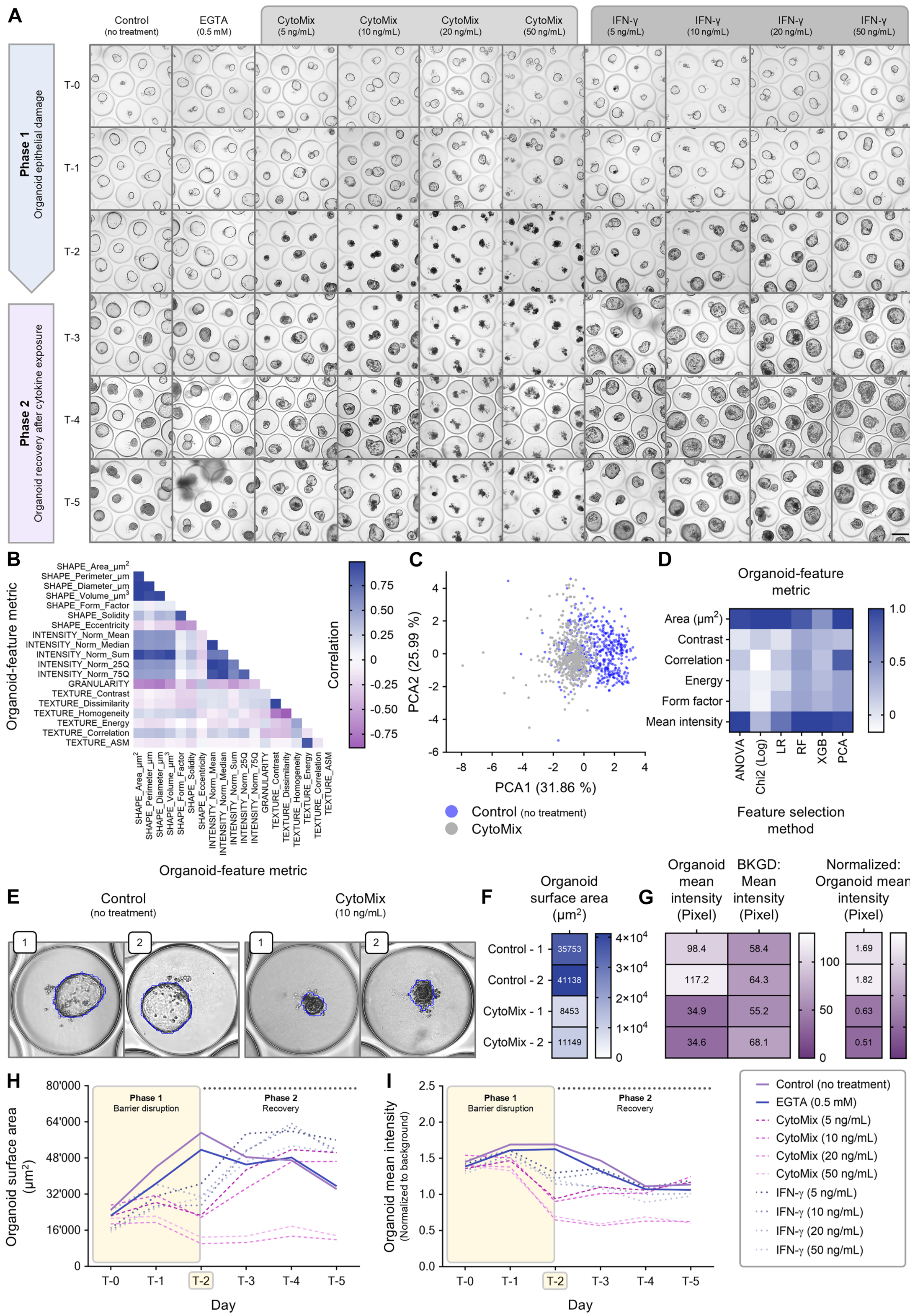

Figure 2. Multimodal profiling of cytokine-induced epithelial disruption and recovery in intestinal organoids

(A) Representative brightfield microscopy images demonstrating morphological changes in intestinal organoids across two experimental phases: phase 1 (epithelial damage induced by CytoMix) and phase 2 (post-treatment recovery). Treatments included control (no treatment), EGTA (0.5 mM) as a chemical barrier-

(legend continued on next page)

phases: (1) epithelial disruption (phase 1, T-0 to T-2) and (2) post-treatment recovery (phase 2, T-3 to T-5). As shown in Figure 2A, organoids treated with CytoMix exhibited a dose-dependent disruption of the epithelial barrier characterized by structural deformation compared to untreated controls. As a chemical control for CytoMix, we used EGTA (ethylene glycol-bis(β-aminoethyl ether)-N,N,N′,N′-tetraacetic acid),[35] a well-characterized calcium chelator known to modulate barrier permeability; however, EGTA at the doses used was not sufficient to consistently alter organoid growth and morphology in this model (Figure 2A). Microscopy analysis revealed distinct CytoMix-induced morphological alterations in the organoids, indicating their responsiveness to the inflammatory stimulus (Figure 2A). The removal of CytoMix resulted in the restoration of normal size and morphology in the organoids, demonstrating their regenerative capacity within this experimental framework. To comprehensively characterize these changes—particularly during the transition from phase 1 to phase 2—precise, high-dimensional quantitative methods at single-organoid resolution are required. Here, we systematically evaluated multiple quantitative metrics of high-dimensional growth dynamics at single-organoid resolution using a microarrayed platform, enabling continuous phenotypic characterization across time (Figures 2B–2D). To decode the complex morphological trajectories, we extracted 19 quantitative features—including morphological, textural, and intensity-based descriptors—from bright-field images using an automated image analysis pipeline built upon SciPy,[36,37] Scikit-image,[38] and OpenCV[39,40] (Figure 2B). This pipeline incorporated a machine learning (ML)-based algorithm for robust organoid segmentation and longitudinal tracking. Correlation analysis of these metrics oriented the selection of a subset of 6 features to capture distinguishing morphological attributes while limiting collinearity in the dataset (Figure 2B). This comprehensive feature set enabled the delineation of unique morphological fingerprints associated with epithelial disruption and subsequent recovery. Dimensionality reduction using principal component analysis (PCA) on these six representative features—including area, contrast, and intensity—effectively captured the variance, distinguishing 203 untreated control organoids from 230 CytoMix-treated organoids (Figure 2C). PCA plots at T-2 to T-4 revealed a clear data separation, with CytoMix-treated organoids exhibiting marked divergence from untreated controls (Figure 2C). This temporal separation emphasizes the capacity of multimodal imaging and feature extraction to trace subtle morphological transitions over time. To prioritize the most informative morphological metrics, we performed comparative feature selection using six independent algorithms, including ANOVA, analysis of variance[41]; Chi2, chi-square test[42]; LR, logistic regression[43]; RF, Random Forest[44]; XGBoost, eXtreme Gradient Boosting[45]; and PCA, principal component analysis[46] (Figure 2D). The resulting heatmap consistently identified two high-ranking features—(1) surface area (μm^2) and (2) intensity—highlighting their robust discriminatory power across treatment conditions and their reproducible ability to distinguish cytokine-induced inflammatory phenotypes (Figure 2D). Representative organoids (Figure 2E) from control and CytoMix-treated groups at T-2 illustrate distinct phenotypic signatures, with disrupted organoids displaying irregular contours, reduced area, and lower intensity. Quantification of the organoid surface area (Figure 2F) confirmed a marked reduction in the CytoMix group, while normalized pixel intensity analysis (Figure 2G) revealed significantly diminished intensity between the organoid and its background, reflecting decreased internal complexity and refractive index homogeneity during the disruption phase. Following the identification and validation of two quantitative features (Figures 2E–2G), these metrics were applied to assess the impact of the dose of pro-inflammatory stimuli by screening across all cytokines (Figures 2H and 2I). Importantly, these metrics should capture the differential effects of various cytokine treatments on epithelial barrier integrity, attenuation of regenerative capacity, and the subsequent recovery phase of the organoid, thereby providing a defined window for potential interventional studies. Accordingly, quantitative analysis using organoid surface area as a metric revealed a marked reduction in response to cytokine exposure, with the effect becoming particularly pronounced at higher CytoMix concentrations ($n \geq 37$ organoids per condition) (Figure 2H). The results were consistent with the microscopic analysis (Figure 2A),

modulating control, CytoMix (a cytokine mixture; concentrations of 5, 10, 20, and 50 ng/mL), and interferon-gamma (IFN-γ; concentrations of 5, 10, 20, and 50 ng/mL). Images captured at sequential time points from T-0 (baseline) through T-5 (end of recovery). Scale bars indicate consistent magnification across treatments and time points. Scale bars: 500 μm.

(B) Correlation (heatmap) analysis was performed on 19 organoid features extracted using SciPy, Scikit-image, and OpenCV.

(C) Principal component analysis (PCA) was conducted on 6 selected features to compare untreated control (blue) and CytoMix-treated (gray; 10 and 20 ng/mL) organoids at time points T-2, T-3, and T-4. The PCA plot illustrates data from 203 untreated control organoids and 230 CytoMix-treated organoids.

(D) A comparative feature importance heatmap, generated using multiple feature selection algorithms, highlights the most discriminative organoid metrics for distinguishing between untreated and CytoMix-treated organoids. ANOVA, analysis of variance; Chi2, chi-square test; LR, logistic regression; RF, Random Forest; XGBoost, eXtreme Gradient Boosting; PCA, principal component analysis. The heatmap displays normalized scores, scaled between the minimum and maximum values for each feature importance method.

(E) Representative organoids with corresponding organoid numbers are shown for untreated and CytoMix-treated (10 ng/mL) conditions at T-2. The blue outline denotes the region of interest (ROI) encompassing each organoid and is inferred using a machine learning (ML)-based segmentation algorithm.

(F) Quantification of organoid area (in μm^2) for the representative samples.

(G) Corresponding mean pixel intensity within the organoid ROI and the background of the image (8-bit scale, where 0 indicates black and 255 indicates white). The normalized organoid mean intensity was calculated by dividing the organoid ROI mean intensity by the mean background intensity.

(H and I) Quantitative analyses of organoid surface area (in μm^2) and normalized mean intensity, respectively, across all treatments and time points. The shaded region highlights the barrier disruption phase (phase 1, T-0 to T-2). Each solid and dashed line represents the average trajectory of individual organoids under different conditions (mean, $n \geq 37$ organoids).

See also Figure S2.

indicating that EGTA had a moderate impact on organoid morphology (Figure 2H). In contrast, cytokines—particularly at concentrations between 5 and 10 ng/mL—induced a strong effect, albeit with evidence of recovery and regeneration following the T-2 period (Figure 2H). Notably, CytoMix at concentrations of 20 ng/mL or higher caused a permanent and non-reversible reduction in the mean intensity metric (Figure 2I). Moreover, organoids exposed to high-dose CytoMix (50 ng/mL) exhibited a marked increase in ethidium homodimer-1 (EthD-1) fluorescence, indicative of elevated cell death, compared to both the control and low-dose (10 ng/mL) CytoMix groups, consistent with the surface area metrics analysis (Figure S2A). Collectively, organoids exposed to 10 ng/mL CytoMix exhibited a pronounced yet reversible reduction in surface area, along with relatively low cytotoxicity, supporting its selection as an appropriate inflammatory stressor to model intestinal epithelial barrier dysfunction and recovery. Normalized mean intensity ($n \geq 37$ organoids per condition) further supported these observations, indicating transient alterations in epithelial density correlating with cytokine concentrations (Figure 2I). Notably, complete morphological recovery was observed during the post-treatment phase, highlighting the intrinsic regenerative capacity of intestinal organoids post-cytokine challenge (Figures 2A, 2H, and 2I). Together, these findings demonstrate that cytokine-induced epithelial injury in intestinal organoids follows a quantifiable and dose-dependent trajectory, with specific morphological features serving as robust proxies for epithelial barrier integrity. The integration of machine vision-based feature extraction and multimodal data analytics enables precise tracking of organoid responses over time, providing a powerful platform for modeling intestinal barrier disruption and recovery. We subsequently applied the same quantitative metrics to healthy organoids to assess their behavior under homeostatic conditions across the distinct stages of organoid development (Figures S2B and S2C). Surface area measurements revealed progressive organoid growth, with individual trajectories displaying variability yet converging toward a consistent mean trend ($n \geq 17$ organoids per condition; Figure S2B). Corresponding mean intensity values, normalized to background levels, showed significant changes over time, reflecting alterations in organoid density and structural complexity (Figure S2C). These findings demonstrate that our quantitative framework is applicable not only for assessing inflammatory responses under challenged conditions but also for monitoring organoid development and ensuring culture quality control. To further integrate the two most informative quantitative features identified previously—organoid surface area and normalized mean intensity—we introduce the "IORS", as a composite longitudinal metric to capture the overall injury-recovery trajectory of individual organoids. Rather than relying on a single endpoint measurement, the IORS is computed by integrating the area under the curve (AUC) of two parameters: the fold-change in organoid surface area (Figures S3A and S3B) and the organoid intensity (Figures S3C and S3D), with the baseline normalized to 1. The final IORS is the scaled sum of these two AUC values, providing a score between 0 and 1 and thereby generating a cumulative readout of epithelial disruption and regenerative capacity (Figure 3A). To validate this scoring approach, we calculated the IORS across the cytokine dose-response conditions described in Figures 2A, 2H, and 2I. As shown in Figure 3B, CytoMix induced a clear and significant reduction in IORS compared with untreated controls, with progressively lower recovery scores observed across increasing CytoMix concentrations. Notably, CytoMix at 10 ng/mL significantly decreased the IORS while still allowing subsequent morphological recovery, supporting its selection as an optimal inflammatory challenge condition for modeling reversible epithelial damage. In contrast, IFN-γ alone produced a less consistent and less dose-dependent reduction in IORS, further indicating that CytoMix more robustly captures epithelial injury and recovery dynamics in these settings. These results validate the IORS as a sensitive composite metric capable of discriminating inflammatory challenge severity and provide a quantitative benchmark for subsequent assessment of bioactive compounds in epithelial recovery assays. Having established that CytoMix 10 ng/mL induces a robust yet reversible epithelial injury phenotype measurable by IORS, we next asked whether this phenotypic response was accompanied by molecular inflammatory programs indicative of intestinal epithelial damage. To gain further insights into the molecular pathways involved in cytokine-induced organoid damage and repair, we performed transcriptomic analysis coupled with gene set enrichment analysis (GSEA) on organoids exposed to CytoMix (10 ng/mL) at the peak of observed morphological changes (Figures 3C and 3D). GSEA analyses revealed significant enrichment in several hallmark inflammatory signaling pathways following exposure to CytoMix (10 ng/mL), with top-ranking pathways, including IL6/JAK/STAT3 signaling, TNF-α signaling via NF-κB, and interferon-gamma response[47,48] (Figure 3D). To assess the translational relevance of this epithelial injury model, we compared the transcriptional responses observed in intestinal epithelial organoids challenged with CytoMix to the gene expression signatures found in intestinal mucosal biopsies obtained from patients with active CD and non-IBD controls[25] (Figure 3E). A comparative scatterplot analysis revealed significant overlaps in inflammatory gene expression patterns between cytokine-treated organoids and active CD patients (Figure 3E). Key inflammatory markers were identified, including genes consistently upregulated (highlighted in red) or downregulated (highlighted in green) across both experimental and clinical conditions (Figure 3E). The expression patterns of several inflammation-associated genes indicate that cytokine-induced morphological changes in organoids capture epithelial inflammatory transcriptional programs that overlap with signatures observed in active CD biopsies,[25] thereby supporting the translational relevance of this epithelial-focused injury-recovery model (Figure 3E).

High-dimensional quantitative phenotyping via the IORS enables precise assessment of therapeutic efficacy in responder organoids

The assessment of multiple organoid parameters—including morphology (shape), granularity, intensity, and size—is essential for accurately characterizing organoid responses,[49,50] as each bioactive ingredient may distinctly modulate specific phenotypic and functional features of organoids.[51–56] Integrating these multidimensional characteristics ensures a comprehensive evaluation of ingredient efficacy by capturing nuanced biological

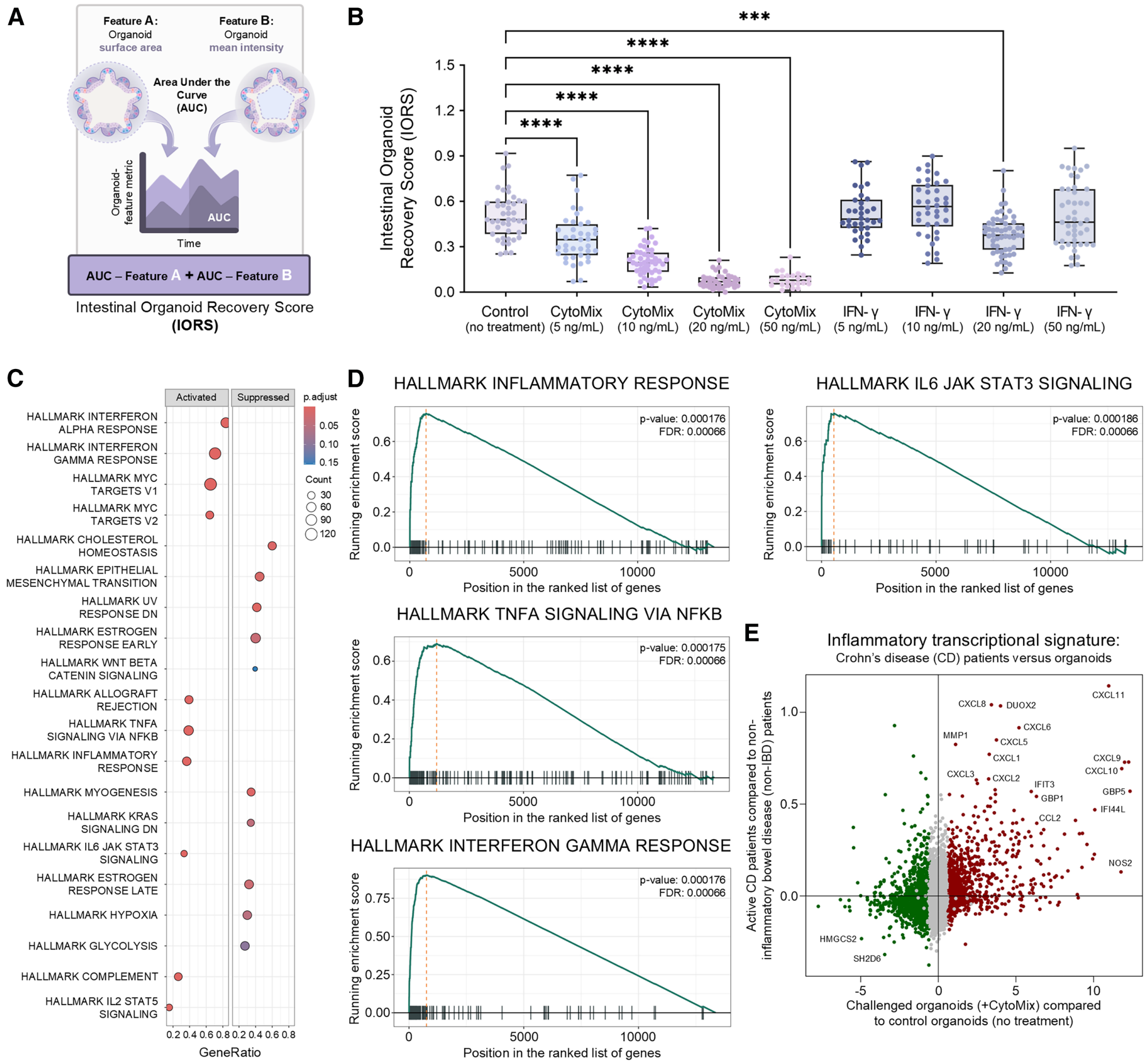

Figure 3. Validation of the intestinal organoid recovery score and cytokine-induced transcriptional reprogramming in intestinal organoids

(A) Schematic representation of the intestinal organoid recovery score (IORS) calculation. IORS integrates the area under the curve (AUC) of two longitudinal image-derived parameters: organoid surface area and organoid mean intensity. The combined AUC values are scaled to generate a composite recovery score reflecting epithelial disruption and regenerative capacity.

(B) Quantification of IORS across untreated control organoids and organoids exposed to increasing concentrations of CytoMix or IFN-γ ($n \geq 23$ organoids per condition). Boxes represent the interquartile range (IQR) with the median indicated; whiskers indicate the minimum to maximum values. Statistical significance is indicated by asterisks (**$p < 0.01$, ***$p < 0.001$, ****$p < 0.0001$). Two-way ANOVA with Bonferroni's post-test was performed using GraphPad Prism.

(C) Gene set enrichment analysis (GSEA) dot plot illustrating the top 20 significantly enriched hallmark signaling pathways—both upregulated and down-regulated—following cytokine exposure (CytoMix, 10 ng/mL) compared to the untreated control at T-2. The dot size represents the number of genes (Count) contributing to each pathway, while the dot color reflects the adjusted p values. The GeneRatio is calculated as the gene count divided by the total number of genes in the respective gene set.

(D) Representative enrichment score plots from GSEA illustrating significant activation of hallmark inflammatory response, IL6/JAK/STAT3 signaling, TNF-α signaling via NF-κB, and interferon-gamma response gene signatures. Statistical significance is indicated by nominal p values and false discovery rate (FDR).

(E) Comparative scatterplot depicting inflammatory transcriptional signatures between cytokine-challenged organoids (CytoMix, 10 ng/mL) relative to untreated controls and gene expression patterns observed in active Crohn's disease (CD) patients versus non-inflammatory bowel disease (non-IBD) controls. Differentially expressed genes are marked in green (downregulated) and red (upregulated), highlighting key inflammatory markers relevant to clinical inflammation status. The genes with the highest differential expression, individually labeled and common to these comparisons, are in the upper right corner.

See also Figure S3.

effects that single-parameter analyses may overlook.[57,58] This approach enables the detection of subtle yet biologically meaningful variations in organoid responses, thereby facilitating the identification of ingredient-specific effects tailored to individual or patient-derived organoid profiles. Inflammatory challenges, as observed with CytoMix (10 ng/mL), influence organoid features such as size, structural integrity, and morphological characteristics, thus providing clear visual and quantitative indicators

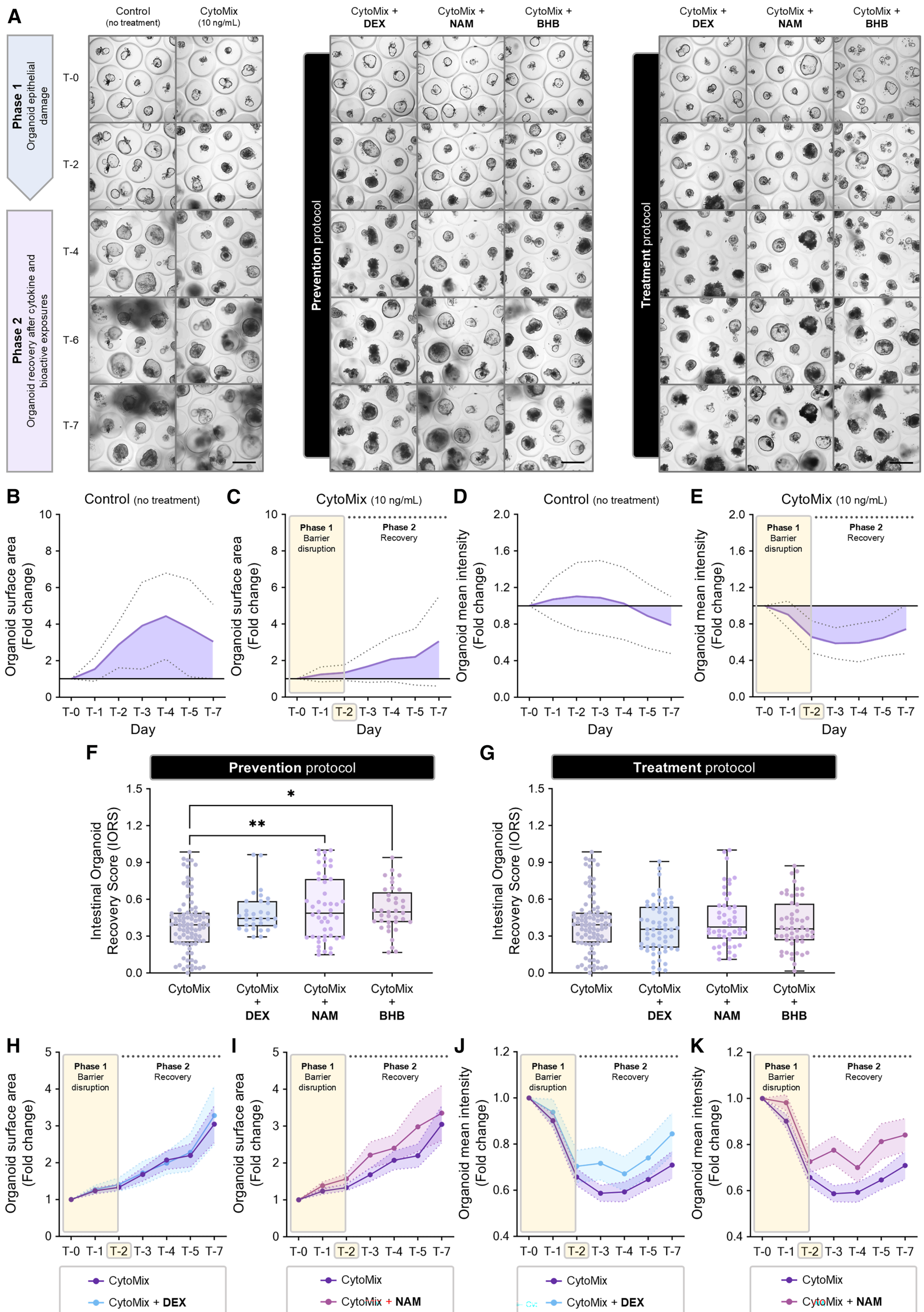

(legend on next page)

for assessing the quality of therapeutic interventions. To further elaborate on this, the current intestinal organoid setup was employed across multiple experimental paradigms to assess whether preventive and therapeutic strategies involving pharmaceutical and nutraceutical interventions can promote recovery from inflammation-induced damage (Figure 4A). Therapeutic efficacy was comprehensively evaluated across two distinct experimental phases—initial cytokine-induced epithelial damage and subsequent recovery—in response to select bioactive compounds: (1) DEX, (2) NAM, and (3) BHB, administered preventively or therapeutically (Figure 4A; Figure S4A). Quantitative metrics, including organoid surface area and normalized mean intensity, supported observations indicating that organoid size and transient changes in epithelial density correlate with cytokine dose, particularly upon exposure to CytoMix (10 ng/mL) (Figures 2H and 2I). Having validated the IORS as a sensitive composite metric for discriminating cytokine-induced epithelial injury severity (Figures 3A and 3B), we next applied this established scoring framework to evaluate whether bioactive interventions could improve epithelial recovery following CytoMix-induced damage. Since IORS integrates the longitudinal area under the curve of both organoid surface area and normalized mean intensity, it provides a more comprehensive assessment of recovery than each parameter alone and reduces the risk of overinterpreting transient or single-feature changes. Accordingly, we applied the validated IORS to quantify organoid recovery dynamics across the full experimental window from T-0 to T-7 in both preventive and treatment protocols. The score was calculated using the same two image-derived parameters—fold change in organoid surface area (Figures 4B and 4C) and organoid mean intensity (Figures 4D and 4E)—as previously validated in the cytokine dose-response setting (Figures 3A and 3B). This application of IORS offers a comprehensive assessment of intestinal organoid recovery, accurately identifying organoids that genuinely respond to diverse treatments and enabling precise tracking of their individual responses over time (Figures 4F and 4G). Application of the IORS revealed that preventive treatments consistently resulted in higher scores compared with CytoMix-only injured controls, highlighting that early initiation of treatment is essential for optimal preservation of intestinal morphology and effective organoid regeneration (Figures 4F and 4G). Among all tested compounds, NAM exhibited the highest IORS, significantly outperforming both the CytoMix-only control—used as the benchmark for evaluating bioactive efficacy—and BHB (Figure 4F). Although BHB showed a promising preventive IORS response, DEX and NAM were prioritized for downstream time-resolved and mechanistic validation because they represent complementary reference conditions. While DEX served as a clinically established anti-inflammatory benchmark, NAM represents the strongest non-steroidal epithelial recovery candidate, with robust IORS improvement and consistent recovery across image-derived parameters. Thus, DEX and NAM enabled us to assess the biological interpretability of IORS across distinct modes of intervention, while BHB remains a promising candidate for dedicated future dose-response and mechanistic studies. Nevertheless, evaluating only a single parameter of organoid growth may overlook this mechanistic diversity, potentially leading to significant misinterpretation of efficacy outcomes as either beneficial or detrimental. Interestingly, time course analyses of single-metric measurements of organoid features revealed distinct kinetic profiles of organoid recovery under therapeutic conditions initiated after epithelial damage, supporting our hypothesis that the outcomes observed in single-metric assessments may depend on the type of ingredient used (Figures 4H–4K). DEX-treated organoids exhibited a gradual yet consistent increase in surface area relative to the CytoMix-only condition (Figure 4H). However, the organoid intensity metric revealed distinct phenotypic differences starting at T-2, diverging from the CytoMix-only condition and maintaining this increasing deviation over subsequent time points until T-7 (Figure 4J). Conversely, NAM-treated organoids exhibited a more rapid initial increase in the surface area metric beginning at T-2 (Figure 4I). Consistently, a similar developmental trend was observed in the intensity metric, though it became evident starting from T-5 compared to the CytoMix-only condition (Figure 4K). Overall, these results underscore the importance of integrative, multi-feature assessments, such as the newly introduced IORS, for enhancing confidence in evaluating diverse

Figure 4. Comparative analysis of preventive and therapeutic interventions on cytokine-induced epithelial disruption and recovery in intestinal organoids

(A) Representative brightfield microscopy images showing morphological changes of organoids across two experimental phases: phase 1 (initial cytokine-induced epithelial damage) and phase 2 (organoid recovery in the presence or absence of interventions). Conditions include control (no treatment), CytoMix (10 ng/mL), and CytoMix plus bioactive compounds dexamethasone (DEX), nicotinamide (NAM), and β-hydroxybutyrate (BHB), applied either as prevention (initiated before epithelial damage) or treatment (initiated after epithelial damage). Images were collected sequentially from baseline (T-0) to endpoint (T-7). Scale bar: 500 μm.

(B–E) Quantitative analyses showing fold-change in organoid surface area (B and C) and normalized mean intensity (D and E), reflecting morphological integrity and epithelial density/structural changes in untreated and CytoMix-challenged conditions over time ($n \geq 90$ organoids). Phase 1 (epithelial damage phase) is highlighted by shaded areas. Solid lines represent means; dashed lines indicate standard deviation (SD). The purple shading highlights the area under the curve (AUC) for each figure, which was used to calculate the intestinal organoid recovery score (IORS) of the respective therapeutic agents.

(F and G) Box-and-whisker plots summarizing the IORS across preventive (F) and treatment (G) intervention groups compared to CytoMix (10 ng/mL) alone ($n \geq$ 31 organoids). Each dot represents an individual organoid. Boxes represent the interquartile range (IQR) with the median indicated; whiskers show the minimum to maximum values. Statistical significance is indicated by asterisks (*$p < 0.05$, **$p < 0.01$). Two-way ANOVA with Bonferroni's post-test was performed using GraphPad Prism.

(H–K) Time course analyses showing fold-change in organoid surface area (H and I) and normalized mean intensity (J and K) for CytoMix-challenged organoids treated with DEX or NAM ($n \geq 41$ organoids). Shaded regions denote barrier disruption phases; lines and shaded bands represent mean values and 95% confidence interval (CI), respectively.

See also Figure S4.

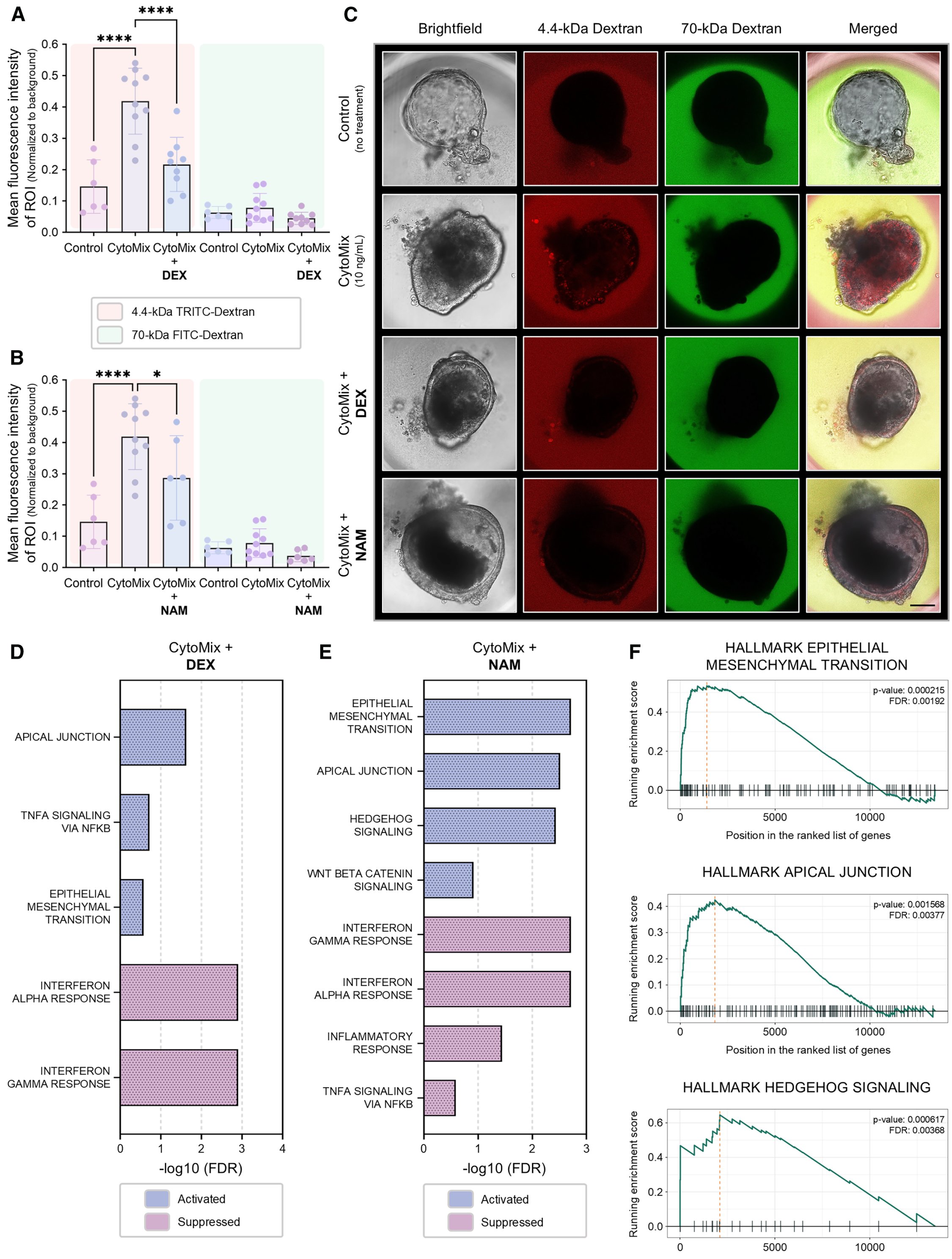

Figure 5. Effects of DEX and NAM on epithelial barrier permeability and transcriptional signaling in cytokine-challenged intestinal organoids

(A and B) Quantitative analysis of barrier permeability in CytoMix-challenged intestinal organoids exposed to 4.4-kDa TRITC-Dextran or 70-kDa FITC-Dextran (large molecule), following CytoMix-induced inflammation (10 ng/mL) alone or with preventive treatment using DEX (A) or NAM (B). Each dot represents an individual organoid. Mean fluorescence intensities were normalized to background, and data are expressed as mean ± standard deviation (SD). Statistical significance is indicated by asterisks (*$p < 0.05$, ****$p < 0.0001$). Two-way ANOVA with Bonferroni's post-test was performed using GraphPad Prism. Untreated controls were included as baseline permeability references for both dextran sizes.

(legend continued on next page)

ingredients with distinct mechanisms of action. By enabling cumulative analysis across multiple phenotypic features within the organoid model, this approach facilitates precise *in vitro* phenotyping. Moreover, these high-dimensional quantitative metrics can discriminate the importance of intervention timing in preserving intestinal epithelium following exposure to different therapeutic agents, such as DEX, NAM, and BHB, thereby providing a high-throughput and rapid readout suitable for the initial screening of potential bioactive ingredients and beneficial therapeutic regimens.

DEX and NAM promote restoration of epithelial barrier function in cytokine-challenged intestinal organoids by modulating transcriptional responses

Although the IORS (Figures 4F and 4G) proved effective for initial screening based on organoid morphological characteristics, further validation of these integrative metrics through functional assays, such as epithelial barrier integrity assessments[57,58] is essential for the selection of bioactive compounds. To investigate the impact of DEX and NAM on epithelial barrier integrity in cytokine-challenged intestinal organoids, barrier permeability was quantitatively assessed using fluorescent dextran uptake assays. To this end, organoids were incubated with 4.4-kDa TRITC-Dextran and 70-kDa FITC-Dextran, following CytoMix (10 ng/mL) treatment alone or in combination with preventive co-treatment (Figures 5A and 5B). The quantitative fluorescence intensity measurements revealed significant alterations in epithelial permeability. CytoMix significantly increased the uptake of small dextran into the lumen compared with control conditions, indicating compromised epithelial barrier integrity. Moreover, the significantly increased permeability to small dextrans, as opposed to the 70-kDa variant, indicates a selective permeability pattern, suggestive of partial epithelial barrier disruption rather than complete loss of integrity (Figures 5A and 5B). Preventive treatment with DEX significantly reduced CytoMix-induced paracellular permeability, restoring it toward control levels (Figure 5A). Similarly, NAM treatment significantly attenuated the CytoMix-induced increase in dextran permeability, particularly demonstrating robust barrier-protective effects for the smaller dextran (Figure 5B). Representative microscopy images corroborated quantitative findings (Figure 5C; Figure S4B). Brightfield images illustrated morphological differences across treatment conditions, with CytoMix-treated organoids appearing structurally compromised. Fluorescent imaging demonstrated enhanced uptake of dextran molecules in CytoMix-only organoids, visualizing increased epithelial permeability. In contrast, preventive treatment groups (CytoMix+DEX and CytoMix+NAM) displayed markedly decreased fluorescent signal intensities, indicating improved barrier function (Figure 5C; Figure S4B). Merged overlays further confirmed permeability differences visually and emphasized the protective impact of DEX and NAM treatments (Figure 5C; Figure S4B). Complementary gene expression analysis followed by GSEA was conducted to elucidate underlying transcriptional changes, revealing distinct pathways modulated by each compound (Figures 5D and 5E). Hallmark signaling pathway analysis identified significant modulations induced by CytoMix in combination with either DEX or NAM. Specifically, DEX treatment prominently suppressed inflammation-related pathways (Figure 5D; Figure S4C), while NAM treatment was notable for modulating epithelial healing-related pathways (Figures 5E and 5F). The detailed GSEA plots (Figure 5F) for NAM-treated organoids revealed significant upregulation of epithelial-mesenchymal transition (EMT)[59,60] and enhanced apical junction integrity,[26] which are critical pathways to promote mucosal healing and restore epithelial barrier function. Moreover, NAM treatment notably influenced the Hedgehog (Hh) signaling pathway,[27,28] underscoring complex transcriptional reprogramming involved in barrier homeostasis (Figure 5F). Collectively, these data demonstrate that preventive treatments with DEX and NAM significantly ameliorate cytokine-induced epithelial barrier dysfunction and promote transcriptional programs associated with epithelial recovery.

DISCUSSION

This study presents a scalable and high-resolution intestinal organoid platform that advances longitudinal modeling of intestinal epithelial dynamics under inflammatory stress. Our approach leverages precise, single-organoid resolution enabled by a machine learning-based segmentation and tracking workflow, which integrates a U-Net convolutional neural network with a fully automated image-analysis pipeline.[61] This approach provides a robust and sensitive tool for dissecting intestinal epithelial damage and regeneration processes central to the pathophysiology of inflammatory intestinal disorders.[6,8,62] Unlike existing static systems, which typically offer only endpoint analyses, our platform uniquely enables time-resolved longitudinal tracking of individual organoid responses. This dynamic capability provides detailed mechanistic insights into the temporal aspects of intestinal epithelial resilience and repair processes, significantly enhancing the resolution and interpretability of inflammatory and regenerative phenotypes. This feature addresses pivotal unresolved questions in inflammation biology, particularly the temporal dynamics of therapeutic interventions and variable regenerative responses across distinct inflammatory contexts.[13,15] Traditional preclinical models, such as 2D cultures and animal models, have

(C) Representative microscopy images depicting intestinal organoids under different treatment conditions: brightfield (morphological structure), fluorescent uptake of small (4.4-kDa) and large (70-kDa) dextrans, and merged overlays for visualization of permeability differences. Treatments include control (no treatment), CytoMix (10 ng/mL) alone, CytoMix plus DEX, and CytoMix plus NAM. Scale bars reflect uniform magnification across all images: 50 μm.

(D and E) Gene set enrichment analysis (GSEA) illustrating significant hallmark signaling pathways activated (blue) or suppressed (purple) by treatment with CytoMix combined with DEX (D) or NAM (E). Bar length indicates significance (-log10 of false discovery rate, FDR).

(F) Detailed enrichment score plots from GSEA analysis showing hallmark pathways significantly modulated by NAM treatments: epithelial-mesenchymal transition (EMT), apical junction integrity, and Hedgehog (Hh) signaling pathway. Significance levels were reported with nominal p values and FDR values.
See also Figure S4.

considerable limitations, including the inability to faithfully replicate human-specific physiological responses and insufficient scalability for high-throughput screening.[13–18] While recent progress has been made in capturing the inflammatory microenvironment of the gastrointestinal tract using 3D organoid systems, key challenges remain.[21,23,24,63–65] These include pronounced inter-organoid variability and persistent issues with reproducibility and scalability, which collectively impede standardization and limit translational potential.[19,66,67] Notably, bulk-scale organoid cultures exhibit pronounced heterogeneity in size, morphology, and differentiation status, complicating data interpretation and reproducibility across experiments.[19,29] To overcome these issues, stringent quality control measures and single-organoid resolution analyses have significantly improved experimental precision and consistency.[51,53,54,68–70] Advances in artificial intelligence (AI)- and ML-based segmentation and tracking systems further reduce phenotypic variability, enhancing the reliability and interpretability of data.[51,68,71–73] Despite these improvements, quantifying organoid growth dynamics under inflammatory stress remains complex due to inherent variability in organoid morphology and growth trajectories. Addressing this requires high-dimensional, quantitative imaging approaches that can precisely characterize morphological features reflective of native epithelial structure and function, ultimately facilitating more accurate assessments of regenerative responses and therapeutic efficacy. The introduction of the IORS represents a significant methodological innovation. By integrating multidimensional metrics, such as organoid surface area and epithelial intensity, the IORS effectively captures multiple aspects of organoid morphology and function, thus overcoming the limitations posed by inherent organoid heterogeneity. This multidimensional approach allows for more precise and reliable discrimination between responders and non-responders to therapeutic interventions, significantly improving experimental robustness and interpretability. Such resolution is crucial for accurately modeling individual-specific responses and can significantly enhance the predictive value of preclinical testing, a cornerstone of personalized medicine and nutrition.[20] Beyond epithelial restitution, future applications could incorporate differentiation readouts to determine whether recovery is accompanied by restoration of homeostatic epithelial lineage composition. Although the image-based metrics and IORS framework could be adapted to conventional dome-based organoid cultures, this would require optimization to address *z* axis variability, organoid overlap, heterogeneous positioning, and reduced tracking efficiency. Thus, the microarrayed format provides practical advantages for standardized, high-throughput, single-organoid-resolved analysis. Importantly, our cytokine-challenged epithelial organoid model captures inflammatory transcriptional programs that overlap with those detected in clinical samples from active CD patients, underscoring its translational relevance.[25] By comparing DEX,[74,75] NAM,[76,77] and BHB,[78,79] the platform revealed distinct recovery-associated responses: DEX primarily suppressed inflammatory signaling,[74,75,80] whereas NAM and BHB supported epithelial barrier and metabolic programs.[77–79,81,82] These findings suggest potential therapeutic implications for relapsing-remitting inflammatory disorders, such as IBD, in which supplementation with NAM, BHB, or their combination could promote epithelial healing to sustain periods of remission and potentially reduce the frequency or severity of subsequent inflammatory episodes. Additionally, by employing high-content transcriptomic profiling coupled with functional assays, such as epithelial permeability, we demonstrate the mechanistic depth achievable with our platform. The distinct transcriptional responses elicited by DEX and NAM—DEX primarily suppresses inflammatory signaling,[75,83,84] and NAM enhances epithelial barrier integrity and metabolic function[77,81]—illustrate the platform's ability to elucidate complex molecular interactions underpinning therapeutic outcomes, aligning closely with existing *in vivo* and clinical data.[74,77,83,85] In conclusion, our epithelial-focused organoid injury–recovery platform addresses key gaps in scalability, resolution, and quantitative assessment of cytokine-induced epithelial barrier disruption and restitution of current preclinical systems. While not capturing the full multicellular complexity of mucosal tissues in IBD, the model provides a standardized, high-throughput platform to investigate epithelial responses to defined inflammatory stressors and to screen interventions that promote barrier recovery. This approach provides a foundation for future studies incorporating patient-derived material, immune or stromal co-cultures, colonic organoids, and microbiota-associated cues to improve physiological relevance and enable patient-informed applications in gastrointestinal inflammatory diseases.

Limitations of the study

The current platform should be interpreted as a reductionist, epithelial-focused organoid assay designed to quantify cytokine-induced epithelial injury and recovery, rather than as a complete model of CD or IBD. Although CytoMix-challenged organoids capture epithelial inflammatory transcriptional programs overlapping with active CD biopsy signatures, the system does not recapitulate the full multicellular and physiological complexity of intestinal inflammation *in vivo*, including immune, stromal, vascular, neuronal, microbial, mechanical, and luminal components.[18,24,86] This study also used organoids derived from non-IBD donor tissue and therefore does not assess patient-to-patient variability or compare diseased and healthy intestinal regions.[87,88] Finally, the current readouts do not directly measure epithelial lineage differentiation or secreted inflammatory mediators.[89] Future studies incorporating patient-derived organoid panels, co-cultures, lineage markers, and media-based cytokine profiling would strengthen physiological and functional interpretation.

RESOURCE AVAILABILITY

Lead contact

Further information and requests for resources and reagents should be directed to and will be fulfilled by the lead contact, Dr. Omid Mashinchian (omid.mashinchian@rd.nestle.com).

Materials availability

This study did not generate unique reagents. Human intestinal organoids were generated and biobanked by DOPPL S.A. under the approved ethical framework described below. Availability of organoid material may be subject to institutional, ethical, legal, and material transfer restrictions.

CellPress
OPEN ACCESS

Data and code availability

- The RNA-seq data generated in this study have been deposited in NCBI's Gene Expression Omnibus GEO: GSE294920 (https://www.ncbi.nlm.nih.gov/geo/query/acc.cgi?acc=GSE294920) and are publicly available as of the date of publication. The public Crohn's disease biopsy dataset used for comparison is available from GEO: GSE52746 (https://www.ncbi.nlm.nih.gov/geo/query/acc.cgi?acc=GSE52746).
- Custom code and associated data used for processing image-extracted numerical data, performing feature selection, and computing the intestinal organoid recovery score (IORS) have been archived on Zenodo and are publicly available at https://doi.org/10.5281/zenodo.21263969. The code is also available from the public DOPPL GitHub repository under public-doppl/iors_pipeline.
- Any additional information required to reanalyze the data reported in this paper is available from the lead contact upon request.

ACKNOWLEDGMENTS

We thank the Nestlé Research community for their valuable discussions and insights regarding the results and their translational applications. We also thank Eline M. van der Beek for the insightful comments on the manuscript, particularly regarding the conceptual framework and translational relevance of the study. This work was supported by funding from Nestlé Research, Société des Produits Nestlé S.A. We acknowledge the non-financial support of the non-profit foundation HTCR, which holds human tissue collections in trust and makes them broadly available for research on an ethical and legal basis.

AUTHOR CONTRIBUTIONS

Conceptualization, M.M., A.R., J. Dervaux, L.F., N.B., M.N., and O.M.; methodology, M.M., A.R., J. Dervaux, L.F., E.P., J. Duc, C.C., S.H.-K., N.B., M.N., and O.M.; investigation, M.M., A.R., J. Dervaux, L.F., C.C., B.C., J.S., S.M., and M.C.; formal analysis, M.M., A.R., J. Dervaux, L.F., E.P., J. Duc, A.K., P.D., M.N., and O.M.; software, E.P., J. Duc, A.K., and P.D.; validation, M.M., A.R., J. Dervaux, L.F., M.C., C.C., B.C., J.S., and S.M.; resources, S.H.-K., M.R.-C.K., P.D., N.B., M.N., and O.M.; data curation, M.M., A.R., J. Dervaux, L.F., E.P., J. Duc, A.K., and P.D.; visualization, M.M., A.R., J. Dervaux, L.F., E.P., J. Duc, M.C., and O.M.; writing – original draft, M.M., A.R., J. Dervaux, L.F., M.N., and O.M.; writing – review and editing, all authors; supervision, N.B., M.N., and O.M.; project administration, N.B., M.N., and O.M.; funding acquisition, M.N. and O.M. Contributions: M.M., A.R., J. Dervaux, and L.F. contributed equally to this work. E.P. and J. Duc contributed equally to computational analysis. N.B., M.N., and O.M. jointly supervised the work.

DECLARATION OF INTERESTS

L.F., M.C., B.C., J.S., S.M., A.K., M.R.-C.K., P.D., M.N., and O.M. are or were employees of Société des Produits Nestlé S.A., Switzerland. M.M., A.R., J. Dervaux, C.C., S.H.-K., and N.B. are or were employees of DOPPL S.A., Switzerland. E.P. and J. Duc are employees of Nexco Analytics Sàrl, Switzerland. Patent protection (EP16199677.2, PCT/EP2017/079651, and US20190367872A1) on the hydrogel microarrayed platform and organoid array technologies described here has been filed, with N.B. and S.H.-K. named as inventors. N.B. and S.H.-K. are shareholders in DOPPL S.A., recently acquired by InSphero AG, which is commercializing those patents.

STAR★METHODS

Detailed methods are provided in the online version of this paper and include the following:

- KEY RESOURCES TABLE
- EXPERIMENTAL MODEL AND STUDY PARTICIPANT DETAILS
  - Human intestinal tissue samples
  - Human intestinal organoids
- METHOD DETAILS
  - Human tissue processing, crypt extraction, and organoid generation
  - Human organoid culture in the microarrayed Gri3D® platform
  - Quantitative imaging pipeline and organoid recovery score assessment using the Gri3D® platform
  - Immunofluorescence staining
  - Live/Dead assay
  - Organoid permeability assay
  - Cytokine-induced epithelial barrier disruption assay
  - Bioactive compound preparation and screening assay
  - RNA extraction
  - Transcriptome profiling: Bulk RNA sequencing (RNA-seq)
  - RNA-seq data analysis
  - Microarray data from patient samples via the Gene Expression Omnibus (GEO: GSE52746)
  - Figure design and schematics
- QUANTIFICATION AND STATISTICAL ANALYSIS

SUPPLEMENTAL INFORMATION

Supplemental information can be found online at https://doi.org/10.1016/j.crmeth.2026.101551.

Received: June 25, 2025
Revised: May 31, 2026
Accepted: July 15, 2026
Published: August 14, 2026

REFERENCES

1. Roda, G., Chien Ng, S., Kotze, P.G., Argollo, M., Panaccione, R., Spinelli, A., Kaser, A., Peyrin-Biroulet, L., and Danese, S. (2020). Crohn's disease. Nat. Rev. Dis. Primers *6*, 22. https://doi.org/10.1038/s41572-020-0156-2.
2. Taku, K., Britta, S., Chen, W.S., Ferrante, M., Shen, B., Bernstein, C.N., Silvio, D., Laurent, P.-B., and Toshifumi, H. (2020). Ulcerative colitis (primer). Nat. Rev. Dis. Primers *6*, 74.
3. Laukoetter, M.G., Nava, P., and Nusrat, A. (2008). Role of the intestinal barrier in inflammatory bowel disease. World J. Gastroenterol. *14*, 401. https://doi.org/10.3748/wjg.14.401.
4. Coskun, M. (2014). Intestinal epithelium in inflammatory bowel disease. Front. Med. *1*, 24. https://doi.org/10.3389/fmed.2014.00024.
5. Vancamelbeke, M., Vanuytsel, T., Farré, R., Verstockt, S., Ferrante, M., Van Assche, G., Rutgeerts, P., Schuit, F., Vermeire, S., Arijs, I., and Cleynen, I. (2017). Genetic and transcriptomic bases of intestinal epithelial barrier dysfunction in inflammatory bowel disease. Inflamm. Bowel Dis. *23*, 1718–1729. https://doi.org/10.1097/mib.0000000000001246.
6. Odenwald, M.A., and Turner, J.R. (2017). The intestinal epithelial barrier: a therapeutic target? Nat. Rev. Gastroenterol. Hepatol. *14*, 9–21. https://doi.org/10.1038/nrgastro.2016.169.
7. Jairath, V., and Feagan, B.G. (2020). Global burden of inflammatory bowel disease. Lancet Gastroenterol. Hepatol. *5*, 2–3. https://doi.org/10.1016/s2468-1253(19)30358-9.
8. Peterson, L.W., and Artis, D. (2014). Intestinal epithelial cells: regulators of barrier function and immune homeostasis. Nat. Rev. Immunol. *14*, 141–153. https://doi.org/10.1038/nri3608.
9. Neurath, M.F., Artis, D., and Becker, C. (2025). The intestinal barrier: a pivotal role in health, inflammation, and cancer. Lancet Gastroenterol. Hepatol. *10*, 573–592.
10. Cho, J.H. (2008). The genetics and immunopathogenesis of inflammatory bowel disease. Nat. Rev. Immunol. *8*, 458–466. https://doi.org/10.1038/nri2340.
11. Lee, S.H., Kwon, J.E., and Cho, M.-L. (2018). Immunological pathogenesis of inflammatory bowel disease. Intest. Res. *16*, 26–42. https://doi.org/10.5217/ir.2018.16.1.26.
12. Baydi, Z., Limami, Y., Khalki, L., Zaid, N., Naya, A., Mtairag, E.M., Oudghiri, M., and Zaid, Y. (2021). An update of research animal models of

inflammatory bowel disease. Sci. World J. *2021*, 7479540. https://doi.org/10.1155/2021/7479540.

13. Rudbaek, J.J., Agrawal, M., Torres, J., Mehandru, S., Colombel, J.-F., and Jess, T. (2024). Deciphering the different phases of preclinical inflammatory bowel disease. Nat. Rev. Gastroenterol. Hepatol. *21*, 86–100. https://doi.org/10.1038/s41575-023-00854-4.
14. Honap, S., Jairath, V., Danese, S., and Peyrin-Biroulet, L. (2024). Navigating the complexities of drug development for inflammatory bowel disease. Nat. Rev. Drug Discov. *23*, 546–562. https://doi.org/10.1038/s41573-024-00953-0.
15. Kelly, C., Sartor, R.B., and Rawls, J.F. (2025). Early subclinical stages of the inflammatory bowel diseases: insights from human and animal studies. Am. J. Physiol. Gastrointest. Liver Physiol. *328*, G17–G31. https://doi.org/10.1152/ajpgi.00252.2024.
16. Antfolk, M., and Jensen, K.B. (2020). A bioengineering perspective on modelling the intestinal epithelial physiology in vitro. Nat. Commun. *11*, 6244. https://doi.org/10.1038/s41467-020-20052-z.
17. Fedi, A., Vitale, C., Ponschin, G., Ayehunie, S., Fato, M., and Scaglione, S. (2021). In vitro models replicating the human intestinal epithelium for absorption and metabolism studies: A systematic review. J. Contr. Release *335*, 247–268. https://doi.org/10.1016/j.jconrel.2021.05.028.
18. Özkan, A., LoGrande, N.T., Feitor, J.F., Goyal, G., and Ingber, D.E. (2024). Intestinal organ chips for disease modelling and personalized medicine. Nat. Rev. Gastroenterol. Hepatol. *21*, 751–773. https://doi.org/10.1038/s41575-024-00968-3.
19. Hofer, M., and Lutolf, M.P. (2021). Engineering organoids. Nat. Rev. Mater. *6*, 402–420. https://doi.org/10.1038/s41578-021-00279-y.
20. Verstegen, M.M.A., Coppes, R.P., Beghin, A., De Coppi, P., Gerli, M.F.M., de Graeff, N., Pan, Q., Saito, Y., Shi, S., Zadpoor, A.A., and van der Laan, L.J.W. (2025). Clinical applications of human organoids. Nat. Med. *31*, 409–421. https://doi.org/10.1038/s41591-024-03489-3.
21. Sarvestani, S.K., Signs, S., Hu, B., Yeu, Y., Feng, H., Ni, Y., Hill, D.R., Fisher, R.C., Ferrandon, S., DeHaan, R.K., et al. (2021). Induced organoids derived from patients with ulcerative colitis recapitulate colitic reactivity. Nat. Commun. *12*, 262. https://doi.org/10.1038/s41467-020-20351-5.
22. Mead, B.E., Hattori, K., Levy, L., Imada, S., Goto, N., Vukovic, M., Sze, D., Kummerlowe, C., Matute, J.D., Duan, J., et al. (2022). Screening for modulators of the cellular composition of gut epithelia via organoid models of intestinal stem cell differentiation. Nat. Biomed. Eng. *6*, 476–494. https://doi.org/10.1038/s41551-022-00863-9.
23. Dennison, T.W., Edgar, R.D., Payne, F., Nayak, K.M., Ross, A.D.B., Cenier, A., Glemas, C., Giachero, F., Foster, A.R., Harris, R., et al. (2024). Patient-derived organoid biobank identifies epigenetic dysregulation of intestinal epithelial MHC-I as a novel mechanism in severe Crohn's Disease. Gut *73*, 1464–1477. https://doi.org/10.1136/gutjnl-2024-332043.
24. Recaldin, T., Steinacher, L., Gjeta, B., Harter, M.F., Adam, L., Kromer, K., Mendes, M.P., Bellavista, M., Nikolaev, M., Lazzaroni, G., et al. (2024). Human organoids with an autologous tissue-resident immune compartment. Nature *633*, 165–173. https://doi.org/10.1038/s41586-024-07791-5.
25. Leal, R.F., Planell, N., Kajekar, R., Lozano, J.J., Ordás, I., Dotti, I., Esteller, M., Masamunt, M.C., Parmar, H., Ricart, E., et al. (2015). Identification of inflammatory mediators in patients with Crohn's disease unresponsive to anti-TNFα therapy. Gut *64*, 233–242. https://doi.org/10.1136/gutjnl-2013-306518.
26. Nguyen, T.P., Otani, T., Tsutsumi, M., Kinoshita, N., Fujiwara, S., Nemoto, T., Fujimori, T., and Furuse, M. (2024). Tight junction membrane proteins regulate the mechanical resistance of the apical junctional complex. J. Cell Biol. *223*, e202307104. https://doi.org/10.1083/jcb.202307104.
27. Buongusto, F., Bernardazzi, C., Yoshimoto, A.N., Nanini, H.F., Coutinho, R.L., Carneiro, A.J.V., Castelo-Branco, M.T., and de Souza, H.S. (2017). Disruption of the Hedgehog signaling pathway in inflammatory bowel disease fosters chronic intestinal inflammation. Clin. Exp. Med. *17*, 351–369. https://doi.org/10.1007/s10238-016-0434-1.
28. Walton, K.D., and Gumucio, D.L. (2021). Hedgehog signaling in intestinal development and homeostasis. Annu. Rev. Physiol. *83*, 359–380. https://doi.org/10.1146/annurev-physiol-031620-094324.
29. Brandenberg, N., Hoehnel, S., Kuttler, F., Homicsko, K., Ceroni, C., Ringel, T., Gjorevski, N., Schwank, G., Coukos, G., Turcatti, G., and Lutolf, M.P. (2020). High-throughput automated organoid culture via stem-cell aggregation in microcavity arrays. Nat. Biomed. Eng. *4*, 863–874. https://doi.org/10.1038/s41551-020-0565-2.
30. Haddad, J.J. (2002). Cytokines and related receptor-mediated signaling pathways. Biochem. Biophys. Res. Commun. *297*, 700–713. https://doi.org/10.1016/s0006-291x(02)02287-8.
31. Turner, M.D., Nedjai, B., Hurst, T., and Pennington, D.J. (2014). Cytokines and chemokines: At the crossroads of cell signalling and inflammatory disease. Biochim. Biophys. Acta *1843*, 2563–2582. https://doi.org/10.1016/j.bbamcr.2014.05.014.
32. Koch, S., and Nusrat, A. (2012). The life and death of epithelia during inflammation: lessons learned from the gut. Annu. Rev. Pathol. *7*, 35–60. https://doi.org/10.1146/annurev-pathol-011811-120905.
33. Cheadle, G.A., Costantini, T.W., Lopez, N., Bansal, V., Eliceiri, B.P., and Coimbra, R. (2013). Enteric glia cells attenuate cytomix-induced intestinal epithelial barrier breakdown. PLoS One *8*, e69042. https://doi.org/10.1371/journal.pone.0069042.
34. Beaurepaire, C., Smyth, D., and McKay, D.M. (2009). Interferon-γ regulation of intestinal epithelial permeability. J. Interferon Cytokine Res. *29*, 133–144. https://doi.org/10.1089/jir.2008.0057.
35. Tria, S., Jimison, L., Hama, A., Bongo, M., and Owens, R. (2013). Sensing of EGTA mediated barrier tissue disruption with an organic transistor. Biosensors *3*, 44–57. https://doi.org/10.3390/bios3010044.
36. Virtanen, P., Gommers, R., Burovski, E., Oliphant, T.E., Weckesser, W., Cournapeau, D., Peterson, P., Reddy, T., Haberland, M., and Wilson, J. (2021). Scipy/Scipy: SciPy 1.6. 0 (Zenodo).
37. Virtanen, P., Gommers, R., Oliphant, T.E., Haberland, M., Reddy, T., Cournapeau, D., Burovski, E., Peterson, P., Weckesser, W., Bright, J., et al. (2020). SciPy 1.0: fundamental algorithms for scientific computing in Python. Nat. Methods *17*, 261–272. https://doi.org/10.1038/s41592-019-0686-2.
38. Van der Walt, S., Schönberger, J.L., Nunez-Iglesias, J., Boulogne, F., Warner, J.D., Yager, N., Gouillart, E., and Yu, T.; scikit-image contributors (2014). scikit-image: image processing in Python. PeerJ *2*, e453. https://doi.org/10.7717/peerj.453.
39. García, G.B., Suarez, O.D., Aranda, J.L.E., Tercero, J.S., Gracia, I.S., and Enano, N.V. (2015). Learning Image Processing with OpenCV (Packt Publishing).
40. Bradski, G. (2000). The opencv library. Dr. Dobb's J. Softw. Tools Prof. Program. *25*, 120–123.
41. Venkatesh, B., and Anuradha, J. (2019). A review of feature selection and its methods. Cybern. Inf. Technol. *19*, 3–26. https://doi.org/10.2478/cait-2019-0001.
42. Liu, H., Zhou, M., and Liu, Q. (2019). An embedded feature selection method for imbalanced data classification. IEEE/CAA J. Autom. Sin. *6*, 703–715. https://doi.org/10.1109/jas.2019.1911447.
43. Wang, J., Wang, H., Nie, F., and Li, X. (2023). Feature selection with multi-class logistic regression. Neurocomputing *543*, 126268. https://doi.org/10.1016/j.neucom.2023.126268.
44. Lefkovits, L., Lefkovits, S., Emerich, S., and Vaida, M.F. (2017). Random Forest Feature Selection Approach for Image Segmentation (SPIE), pp. 227–231.
45. Thenmozhi, T., and Helen, R. (2022). Feature selection using extreme gradient boosting Bayesian optimization to upgrade the classification performance of motor imagery signals for BCI. J. Neurosci. Methods *366*, 109425. https://doi.org/10.1016/j.jneumeth.2021.109425.
46. Song, F., Guo, Z., and Mei, D. (2010). Feature Selection Using Principal Component Analysis (IEEE), pp. 27–30.

47. Yeung, Y.T., Aziz, F., Guerrero-Castilla, A., and Arguelles, S. (2018). Signaling pathways in inflammation and anti-inflammatory therapies. Curr. Pharm. Des. *24*, 1449–1484. https://doi.org/10.2174/1381612824666180327165604.

48. Agirman, G., Yu, K.B., and Hsiao, E.Y. (2021). Signaling inflammation across the gut-brain axis. Science *374*, 1087–1092. https://doi.org/10.1126/science.abi6087.

49. Karolak, A., Poonja, S., and Rejniak, K.A. (2019). Morphophenotypic classification of tumor organoids as an indicator of drug exposure and penetration potential. PLoS Comput. Biol. *15*, e1007214. https://doi.org/10.1371/journal.pcbi.1007214.

50. Montes-Olivas, S., Legge, D., Lund, A., Fletcher, A.G., Williams, A.C., Marucci, L., and Homer, M. (2023). In-silico and in-vitro morphometric analysis of intestinal organoids. PLoS Comput. Biol. *19*, e1011386. https://doi.org/10.1371/journal.pcbi.1011386.

51. Gu, Y., Zhang, W., Wu, X., Zhang, Y., Xu, K., and Su, J. (2023). Organoid assessment technologies. Clin. Transl. Med. *13*, e1499. https://doi.org/10.1002/ctm2.1499.

52. Ong, H.T., Karatas, E., Poquillon, T., et al. (2025). Digitalized organoids: integrated pipeline for high-speed 3D analysis of organoid structures using multilevel segmentation and cellular topology. Nat. Methods *22*, 1343–1354. https://doi.org/10.1038/s41592-025-02685-4.

53. Bukas, C., Subramanian, H., See, F., Steinchen, C., Ezhov, I., Boosarpu, G., Asgharpour, S., Burgstaller, G., Lehmann, M., Kofler, F., and Piraud, M. (2024). MultiOrg: a multi-rater organoid-detection dataset. Proceedings of the Advances in Neural Information Processing Systems *37*, 95808–95839. https://doi.org/10.52202/079017-3036.

54. Bai, L., Wu, Y., Li, G., Zhang, W., Zhang, H., and Su, J. (2024). AI-enabled organoids: construction, analysis, and application. Bioact. Mater. *31*, 525–548. https://doi.org/10.1016/j.bioactmat.2023.09.005.

55. Cheng, C.-W., Biton, M., Haber, A.L., Gunduz, N., Eng, G., Gaynor, L.T., Tripathi, S., Calibasi-Kocal, G., Rickelt, S., Butty, V.L., et al. (2019). Ketone body signaling mediates intestinal stem cell homeostasis and adaptation to diet. Cell *178*, 1115–1131.e15. https://doi.org/10.1016/j.cell.2019.07.048.

56. Imada, S., Khawaled, S., Shin, H., Meckelmann, S.W., Whittaker, C.A., Corrêa, R.O., Alquati, C., Lu, Y., Tie, G., Pradhan, D., et al. (2024). Short-term post-fast refeeding enhances intestinal stemness via polyamines. Nature *633*, 895–904. https://doi.org/10.1038/s41586-024-07840-z.

57. Tagesson, C., Sjödahl, R., and Thorén, B. (1978). Passage of molecules through the wall of the gastrointestinal tract: I. A simple experimental model. Scand. J. Gastroenterol. *13*, 519–524. https://doi.org/10.3109/00365527809181758.

58. Snelson, M., Vanuytsel, T., and Marques, F.Z. (2024). Breaking the barrier: The role of gut epithelial permeability in the pathogenesis of hypertension. Curr. Hypertens. Rep. *26*, 369–380. https://doi.org/10.1007/s11906-024-01307-2.

59. Jiang, H., Shen, J., and Ran, Z. (2018). Epithelial–mesenchymal transition in Crohn's disease. Mucosal Immunol. *11*, 294–303. https://doi.org/10.1038/mi.2017.107.

60. Youssef, K.K., and Nieto, M.A. (2024). Epithelial–mesenchymal transition in tissue repair and degeneration. Nat. Rev. Mol. Cell Biol. *25*, 720–739. https://doi.org/10.1038/s41580-024-00733-z.

61. Ronneberger, O., Fischer, P., and Brox, T. (2015). U-net: Convolutional Networks for Biomedical Image Segmentation (Springer), pp. 234–241.

62. Koch, S., and Nusrat, A. (2009). Dynamic regulation of epithelial cell fate and barrier function by intercellular junctions. Ann. N. Y. Acad. Sci. *1165*, 220–227. https://doi.org/10.1111/j.1749-6632.2009.04025.x.

63. Daghero, H., Doffe, F., Varela, B., Yozzi, V., Verdes, J.M., Crispo, M., Bollati-Fogolín, M., and Pagotto, R. (2022). Jejunum-derived NF-κB reporter organoids as 3D models for the study of TNF-alpha-induced inflammation. Sci. Rep. *12*, 14425. https://doi.org/10.1038/s41598-022-18556-3.

64. Pavlidis, P., Tsakmaki, A., Treveil, A., Li, K., Cozzetto, D., Yang, F., Niazi, U., Hayee, B.H., Saqi, M., Friedman, J., et al. (2022). Cytokine responsive networks in human colonic epithelial organoids unveil a molecular classification of inflammatory bowel disease. Cell Rep. *40*, 111439. https://doi.org/10.1016/j.celrep.2022.111439.

65. Santos, A.J.M., van Unen, V., Lin, Z., Chirieleison, S.M., Ha, N., Batish, A., Chan, J.E., Cedano, J., Zhang, E.T., Mu, Q., et al. (2024). A human autoimmune organoid model reveals IL-7 function in coeliac disease. Nature *632*, 401–410. https://doi.org/10.1038/s41586-024-07716-2.

66. Zhao, Z., Chen, X., Dowbaj, A.M., Sljukic, A., Bratlie, K., Lin, L., Fong, E.L.S., Balachander, G.M., Chen, Z., Soragni, A., et al. (2022). Organoids. Nat. Rev. Methods Primers *2*, 94. https://doi.org/10.1038/s43586-022-00174-y.

67. Heinzelmann, E., Piraino, F., Costa, M., Roch, A., Norkin, M., Garnier, V., Homicsko, K., and Brandenberg, N. (2024). iPSC-derived and Patient-Derived Organoids: Applications and challenges in scalability and reproducibility as pre-clinical models. Curr. Res. Toxicol. *7*, 100197. https://doi.org/10.1016/j.crtox.2024.100197.

68. Matthews, J.M., Schuster, B., Kashaf, S.S., Liu, P., Ben-Yishay, R., Ishay-Ronen, D., Izumchenko, E., Shen, L., Weber, C.R., Bielski, M., et al. (2022). OrganoID: A versatile deep learning platform for tracking and analysis of single-organoid dynamics. PLoS Comput. Biol. *18*, e1010584. https://doi.org/10.1371/journal.pcbi.1010584.

69. Tebon, P.J., Wang, B., Markowitz, A.L., Davarifar, A., Tsai, B.L., Krawczuk, P., Gonzalez, A.E., Sartini, S., Murray, G.F., Nguyen, H.T.L., et al. (2023). Drug screening at single-organoid resolution via bioprinting and interferometry. Nat. Commun. *14*, 3168. https://doi.org/10.1038/s41467-023-38832-8.

70. Maramraju, S., Kowalczewski, A., Kaza, A., Liu, X., Singaraju, J.P., Albert, M.V., Ma, Z., and Yang, H. (2024). AI-organoid integrated systems for biomedical studies and applications. Bioeng. Transl. Med. *9*, e10641. https://doi.org/10.1002/btm2.10641.

71. Huang, K., Li, M., Li, Q., Chen, Z., Zhang, Y., and Gu, Z. (2024). Image-based profiling and deep learning reveal morphological heterogeneity of colorectal cancer organoids. Comput. Biol. Med. *173*, 108322. https://doi.org/10.1016/j.compbiomed.2024.108322.

72. Zhou, J., Fu, Z., Ni, X., Luo, Q., and Yang, G. (2024). ISCO: Intelligent Framework for Accurate Segmentation and Comparative Analysis of Organoids. Preprint at bioRxiv. https://doi.org/10.1101/2024.12.24.630244.

73. Takagi, K., Takagi, M., Hiyama, G., and Goda, K. (2024). A deep-learning model for characterizing tumor heterogeneity using patient-derived organoids. Sci. Rep. *14*, 22769. https://doi.org/10.1038/s41598-024-73725-w.

74. Madamsetty, V.S., Mohammadinejad, R., Uzieliene, I., Nabavi, N., Dehshahri, A., García-Couce, J., Tavakol, S., Moghassemi, S., Dadashzadeh, A., Makvandi, P., et al. (2022). Dexamethasone: insights into pharmacological aspects, therapeutic mechanisms, and delivery systems. ACS Biomater. Sci. Eng. *8*, 1763–1790. https://doi.org/10.1021/acsbiomaterials.2c00026.

75. Lu, L., Lu, J., Yu, Y., and Claud, E. (2021). Necrotizing enterocolitis intestinal barrier function protection by antenatal dexamethasone and surfactant-D in a rat model. Pediatr. Res. *90*, 768–775. https://doi.org/10.1038/s41390-020-01334-0.

76. Yoshino, J., Baur, J.A., and Imai, S.-i. (2018). NAD+ intermediates: the biology and therapeutic potential of NMN and NR. Cell Metab. *27*, 513–528. https://doi.org/10.1016/j.cmet.2017.11.002.

77. Chen, C., Yan, W., Tao, M., and Fu, Y. (2023). NAD+ metabolism and immune regulation: new approaches to inflammatory bowel disease therapies. Antioxidants *12*, 1230. https://doi.org/10.3390/antiox12061230.

78. Wang, T., Zhuang, Y., Yu, C., Wang, Z., Liu, Y., Xu, Q., Liu, K., and Li, Y. (2024). D-beta-hydroxybutyrate up-regulates Claudin-1 and alleviates the intestinal hyperpermeability in lipopolysaccharide-treated mice. Tissue Cell *87*, 102343. https://doi.org/10.1016/j.tice.2024.102343.

79. Huang, C., Wang, J., Liu, H., Huang, R., Yan, X., Song, M., Tan, G., and Zhi, F. (2022). Ketone body β-hydroxybutyrate ameliorates colitis by promoting

M2 macrophage polarization through the STAT6-dependent signaling pathway. BMC Med. *20*, 148. https://doi.org/10.1186/s12916-022-02352-x.

80. Xu, P., Elizalde, M., Masclee, A., Pierik, M., and Jonkers, D. (2021). Corticosteroid enhances epithelial barrier function in intestinal organoids derived from patients with Crohn's disease. J. Mol. Med. *99*, 805–815. https://doi.org/10.1007/s00109-021-02045-7.
81. Ling, C., Versloot, C.J., Kvissberg, M.E.A., Hu, G., Swain, N., Horcas-Nieto, J.M., Miraglia, E., Thind, M.K., Farooqui, A., and Gerding, A. (2023). Rebalancing of mitochondrial homeostasis through an NAD+-SIRT1 pathway preserves intestinal barrier function in severe malnutrition. EBioMedicine *96*, 104809.
82. Shay, J.E.S., and Yilmaz, Ö.H. (2025). Dietary and metabolic effects on intestinal stem cells in health and disease. Nat. Rev. Gastroenterol. Hepatol. *22*, 23–38. https://doi.org/10.1038/s41575-024-00980-7.
83. Kumar, A., Husain, N., Anbazhagan, A.N., Jayawardena, D., Priyamvada, S., Singhal, M., Jain, C., Kaur, P., Guzman, G., and Saksena, S. (2024). Dexamethasone Upregulates the Expression of the Human SLC26A3 (DRA, D own-R egulated in A denoma) Transporter (an IBD Susceptibility Gene) in Intestinal Epithelial Cells and Attenuates Gut Inflammation. Inflamm. Bowel Dis. *31*, 625–635.
84. Robinson, J.M., Turkington, S., Abey, S.A., Kenea, N., and Henderson, W.A. (2019). Differential gene expression and gene-set enrichment analysis in Caco-2 monolayers during a 30-day timeline with Dexamethasone exposure: A data modeling approach to understanding culture age as covariate for differential expression in a non-renewing epithelial monolayer using a gene ontology-defined 250-plex Nanostring probe panel. Tissue Barriers *7*, e1651597. https://doi.org/10.1080/21688370.2019.1651597.
85. Villar, J., Ferrando, C., Martínez, D., Ambrós, A., Muñoz, T., Soler, J.A., Aguilar, G., Alba, F., González-Higueras, E., Conesa, L.A., et al. (2020). Dexamethasone treatment for the acute respiratory distress syndrome: a multicentre, randomised controlled trial. Lancet Respir. Med. *8*, 267–276. https://doi.org/10.1016/s2213-2600(19)30417-5.
86. Arnauts, K., Verstockt, B., Ramalho, A.S., Vermeire, S., Verfaillie, C., and Ferrante, M. (2020). Ex vivo mimicking of inflammation in organoids derived from patients with ulcerative colitis. Gastroenterology *159*, 1564–1567. https://doi.org/10.1053/j.gastro.2020.05.064.
87. Karakasheva, T.A., Zhou, Y., Xie, H.M., Soto, G.E., Johnson, T.D., Stoltz, M.A., Roach, D.M., Nema, N., Umeweni, C.N., Naughton, K., et al. (2023). Patient-derived colonoids from disease-spared tissue retain inflammatory bowel disease-specific transcriptomic signatures. Gastro Hep Adv. *2*, 830–842. https://doi.org/10.1016/j.gastha.2023.05.003.
88. Arnauts, K., Verstockt, B., Sabino, J., Vermeire, S., Verfaillie, C., and Ferrante, M. (2020). OP11 exposure to an inflammatory mix re-induces inflammation in organoids of ulcerative colitis patients, independent of the inflammatory state of the tissue of origin. J. Crohns Colitis *14*, S011–S012. https://doi.org/10.1093/ecco-jcc/jjz203.010.
89. Capeling, M.M., Chen, B., Aliar, K., Penna, E., Lopez, V.I., Foo, C., Rost, S., Holokai, L., Tong, X., and Phillips, D. (2026). Dictionary of human intestinal organoid responses to secreted niche factors at single cell resolution. Nat. Commun. *17*, 1527.
90. Kim, D., Paggi, J.M., Park, C., Bennett, C., and Salzberg, S.L. (2019). Graph-based genome alignment and genotyping with HISAT2 and HISAT-genotype. Nat. Biotechnol. *37*, 907–915. https://doi.org/10.1038/s41587-019-0201-4.
91. Liao, Y., Smyth, G.K., and Shi, W. (2014). featureCounts: an efficient general purpose program for assigning sequence reads to genomic features. Bioinformatics *30*, 923–930. https://doi.org/10.1093/bioinformatics/btt656.
92. Ritchie, M.E., Phipson, B., Wu, D., Hu, Y., Law, C.W., Shi, W., and Smyth, G.K. (2015). limma powers differential expression analyses for RNA-sequencing and microarray studies. Nucleic Acids Res. *43*, e47. https://doi.org/10.1093/nar/gkv007.
93. Thasler, W.E., Weiss, T.S., Schillhorn, K., Stoll, P.-T., Irrgang, B., and Jauch, K.-W. (2003). Charitable state-controlled foundation human tissue and cell research: ethic and legal aspects in the supply of surgically removed human tissue for research in the academic and commercial sector in Germany. Cell Tissue Bank. *4*, 49–56. https://doi.org/10.1023/A:1026392429112.
94. Fujii, M., Matano, M., Toshimitsu, K., Takano, A., Mikami, Y., Nishikori, S., Sugimoto, S., and Sato, T. (2018). Human intestinal organoids maintain self-renewal capacity and cellular diversity in niche-inspired culture condition. Cell Stem Cell *23*, 787–793.e6. https://doi.org/10.1016/j.stem.2018.11.016.
95. Anstett, V., Heinzelmann, E., Piraino, F., Roch, A., Chrisnandy, A., Norkin, M., Garnier, V., Homicsko, K., Hoehnel-Ka, S., and Brandenberg, N. (2025). In Vitro Evaluation of the Safety and Efficacy of Cibisatamab Using Adult Stem Cell-Derived Organoids and Colorectal Cancer Spheroids. Cancers *17*, 291. https://doi.org/10.3390/cancers17020291.
96. Law, C.W., Chen, Y., Shi, W., and Smyth, G.K. (2014). voom: Precision weights unlock linear model analysis tools for RNA-seq read counts. Genome Biol. *15*, R29. https://doi.org/10.1186/gb-2014-15-2-r29.
97. Benjamini, Y., and Hochberg, Y. (1995). Controlling the false discovery rate: a practical and powerful approach to multiple testing. J. Roy. Stat. Soc. B Stat. Methodol. *57*, 289–300. https://doi.org/10.1111/j.2517-6161.1995.tb02031.x.

CellPress
OPEN ACCESS

STAR★METHODS

KEY RESOURCES TABLE

REAGENT or RESOURCE	SOURCE	IDENTIFIER
Antibodies		
Anti-Ki-67 antibody	Invitrogen	Cat# MA5-14520; RRID: AB_10979488
Anti-E-cadherin antibody	Abcam	Cat# ab231303; RRID: AB_2923285
Secondary antibodies for immunofluorescence	Thermo Fisher Scientific	Cat# A31571; RRID: AB_162542, Cat# A31573; RRID: AB_2536183
Biological samples		
Human small intestinal biopsy specimens	Human Tissue and Cell Research Foundation, LMU Munich, Germany	Ethics approvals: LMU Faculty of Medicine 025-12; Bavarian State Medical Association 11142
Human intestinal organoids derived from small intestinal biopsies	DOPPL S.A., Lausanne, Switzerland	CER-VD AO_2021-00076, CER-VD 2024-02364
Chemicals, peptides, and recombinant proteins		
Matrigel™	Corning	Cat# 356231
Collagenase I	Thermo Fisher Scientific	Cat# 17018029
Collagenase II	Thermo Fisher Scientific	Cat# 17101015
Hyaluronidase	Sigma-Aldrich	Cat# H3884
TrypLE	Thermo Fisher Scientific	Cat# 12605028
Advanced DMEM/F12	Thermo Fisher Scientific	Cat# 12634010
GlutaMAX™	Thermo Fisher Scientific	Cat# 35050-038
HEPES	Thermo Fisher Scientific	Cat# 15630-056
Penicillin/Streptomycin	Thermo Fisher Scientific	Cat# 15140-122
B-27 Supplement without vitamin A	Thermo Fisher Scientific	Cat# 12587-001
Phosphate-buffered saline (PBS)	Thermo Fisher Scientific	Cat# 10010056
Bovine serum albumin (BSA)	Thermo Fisher Scientific	Cat# 15260-037
4% paraformaldehyde	Thermo Fisher Scientific	Cat# J61899
Triton X-100	Sigma-Aldrich	Cat# T8787
Hanks' Balanced Salt Solution (HBSS)	Thermo Fisher Scientific	Cat# 14025050
DAPI	Thermo Fisher Scientific	Cat# 62248
Phalloidin	Abcam	Cat# ab176753
Recombinant human TNF-α	Thermo Fisher Scientific	Cat# 300-01A
Recombinant human IL-1β	Thermo Fisher Scientific	Cat# 200-01B
Recombinant human IFN-γ	Thermo Fisher Scientific	Cat# 300-02
EGTA	Sigma-Aldrich	Cat# E3889
Dexamethasone (DEX)	Sigma-Aldrich	Cat# D4902
Nicotinamide/niacinamide (NAM)	DSM-Firmenich	Cat# 0429449
β-hydroxybutyrate magnesium salt (BHB; 3HB-Mg)	Osaka Gas	Identifier: 3HB-Mg
Dimethyl sulfoxide (DMSO)	Sigma-Aldrich	Cat# D2438
4.4-kDa TRITC–Dextran	Sigma-Aldrich	Cat# 46944
70-kDa FITC–Dextran	Sigma-Aldrich	Cat# 90718
Critical commercial assays		
LIVE/DEAD™ Viability/Cytotoxicity Kit	Thermo Fisher Scientific	Cat# L3224
LBE lysis buffer	Beckman Coulter	Cat# C39467
Agencourt RNAdvance Tissue Kit	Beckman Coulter	Cat# A32649
Quant-iT™ RiboGreen RNA Assay Kit	Invitrogen	Cat# R11490

(*Continued on next page*)

Continued

REAGENT or RESOURCE	SOURCE	IDENTIFIER
Standard Sensitivity RNA Analysis Kit	Agilent Technologies	Cat# DNF-471-0500
Illumina Stranded mRNA Library Prep Kit	Illumina	Cat# 20040534
Deposited data		
RNA-seq data generated in this study	NCBI Gene Expression Omnibus	GEO: GSE294920
Crohn's disease biopsy microarray dataset	NCBI Gene Expression Omnibus	GEO: GSE52746
Human reference genome hg19	UCSC Genome Browser/Genome Reference Consortium	hg19
MSigDB Hallmark gene sets	Molecular Signatures Database	MSigDB Hallmark collection
Software and algorithms		
Custom IORS image-analysis pipeline	This paper/DOPPL GitHub/Zenodo	GitHub: public-doppl/iors_pipeline; DOI: https://doi.org/10.5281/zenodo.21263969
U-Net-based organoid segmentation algorithm	Ronneberger et al.,[61] and this paper	N/A
SciPy	Virtanen et al.[36,37]	v1.11; https://scipy.org/
scikit-image	van der Walt et al.[38]	v0.22; https://scikit-image.org/
OpenCV	Bradski et al.[39,40]	v4.8; https://opencv.org/
CellProfiler	Broad Institute	v4.2.5; https://cellprofiler.org/
Tidyverse	R package	Version used with R v4.3.1; https://tidyverse.org/
R	R Foundation for Statistical Computing	v4.2.1/v4.3.1; https://www.r-project.org/
HISAT2	Kim et al.[90]	v2.2.1
featureCounts	Liao et al.[91]	v2.0.1
limma	Bioconductor; Ritchie et al.[92]	v3.52.3; https://bioconductor.org/
clusterProfiler	Bioconductor	v4.2.2; https://bioconductor.org/
GraphPad Prism	GraphPad Software	v9.3.0; https://www.graphpad.com/
CorelDraw Graphics Suite ×8	Corel Corporation	v18.0.0.448; https://www.coreldraw.com/
BioRender	BioRender	https://www.biorender.com/
Other		
Gri3D® microarrayed hydrogel platform	SUN bioscience. Brandenberg et al.[29]	Cat# Gri3D-96P-S-96-500
Nikon Ti-2 microscope	Nikon	N/A
Zeiss LSM700 confocal microscope	Zeiss	N/A
IN Cell Analyzer	GE Healthcare	N/A
FastPrep-24™ instrument	MP Biomedicals	N/A
SpectraMax® F1 Microplate Reader	Molecular Devices	N/A
Fragment Analyzer 96-capillary system	Agilent Technologies	N/A
Illumina NextSeq 2000 platform	Illumina	N/A

EXPERIMENTAL MODEL AND STUDY PARTICIPANT DETAILS

Human intestinal tissue samples

Human small intestinal biopsy specimens and annotated data were obtained and processed within the framework of the non-profit Human Tissue and Cell Research (HTCR) Foundation at Ludwig-Maximilians-University (LMU), Munich, Germany. All tissue collections were performed in accordance with the HTCR framework, which includes written informed consent from all donors and approval by the Ethics Committee of the LMU Faculty of Medicine (approval number 025-12) and the Bavarian State Medical Association (approval number 11142). The legal and ethical compliance of the HTCR Foundation has been described previously.[93] Donor age, sex, and gender metadata were not available to the authors for the de-identified HTCR biopsy specimens; therefore, the influence of age, sex, or gender on the results could not be assessed.

Human intestinal organoids

Human intestinal organoid generation and biobanking from patient-derived biopsy material were approved by the Cantonal Ethics Committee (CER-VD AO_2021-00076) and performed by DOPPL S.A. (Lausanne, Switzerland). The reuse of organoids from this

biobank for the activities described here was approved by CER-VD (approval number 2024–02364). Organoids were derived from human small intestinal biopsy specimens and maintained in customized intestinal organoid culture medium as described below.

METHOD DETAILS

Human tissue processing, crypt extraction, and organoid generation

Briefly, biopsy specimens were thoroughly rinsed in phosphate-buffered saline (PBS; 10010056, Thermo Fisher Scientific), and the underlying muscle layers were meticulously dissected using surgical scissors. The epithelial tissue was then minced into ~1-mm fragments and subjected to enzymatic digestion with collagenase I (17018029, Thermo Fisher Scientific), collagenase II (17101015, Thermo Fisher Scientific), and hyaluronidase (H3884, Sigma-Aldrich) for 30 min at 37°C. Following additional washes, isolated crypts were embedded in 15 μL droplets of Matrigel™ (356231, Corning), positioned centrally within wells of a 96-well plate. Each drop was subsequently overlaid with 200 μL of a customized intestinal organoid culture medium, developed in-house at DOPPL S.A. This formulation was adapted from a previously published protocol[94] and consisted of advanced DMEM/F12 supplemented with GlutaMAX™ (35050-038, Thermo Fisher Scientific), HEPES (15630-056, Thermo Fisher Scientific), penicillin/streptomycin (15140-122, Thermo Fisher Scientific), and B-27 Supplement without Vitamin A (12587-001, Thermo Fisher Scientific).

Human organoid culture in the microarrayed Gri3D® platform

Organoids were cultured for seven days in Matrigel™ domes. Subsequently, organoids were harvested, enzymatically dissociated into single cells using TrypLE (12605028, Thermo Fisher Scientific) for 10 min at 37°C, and resuspended in an organoid culture medium. Single cells were seeded into microwells at a density of 150 cells per well in a 50 μL volume. Plates (Gri3D® - SUN bioscience) were incubated for 20 min to facilitate cell sedimentation into the microcavities, after which 150 μL of the medium was gently added via the pipetting port. The Gri3D® platform was incubated under static conditions at 37°C with 5% CO_2 for 72 h to facilitate the initial formation of three-dimensional intestinal organoid structures. Subsequently, the cultures were maintained in the intestinal organoid medium for up to 12 days, with the medium refreshed every 2–3 days, to support organoid expansion and differentiation. For downstream analyses, such as transcriptome profiling, each well of the Gri3D® platform was collected individually on day 6 (T-2) or day 11 (T-8). Four wells of the same condition were collected separately and analyzed as quadruplicates for transcriptome profiling. Samples were lysed using LBE lysis buffer (C39467, Beckman Coulter) and vortexed for 1 min. Lysates were stored at −80°C before RNA extraction and subsequent analysis.

Quantitative imaging pipeline and organoid recovery score assessment using the Gri3D® platform

Organoids were imaged daily in brightfield using a Nikon Ti-2 microscope. Longitudinal tracking and feature extraction were performed using an image analysis pipeline.[95] This pipeline employs a convolutional neural network based on the U-Net architecture[61] for organoid segmentation. In instances where multiple objects were detected within a single microwell, a filtering step retained only the largest object. Organoids that were not detected at one or more time points were excluded from the analysis. In total, approximately 20 organoids per well were analyzed out of 73 available microcavities. Detected organoids in the Gri3D® platform were then tracked individually over time, and a broad set of morphological features was extracted using the SciPy,[36,37] Scikit-image,[38] and OpenCV[39,40] libraries. All intensity-related metrics were normalized to the image background mean intensity to ensure that global differences in illumination have no impact on the results. Subsequently, a comprehensive feature selection analysis was conducted on these 19 metrics. This analysis encompassed organoids subjected to untreated control conditions as well as CytoMix treatments at concentrations of 10 ng/mL and 20 ng/mL. The evaluation was performed at specific time points corresponding to the application of the challenge (T-2, T-3, T-4). To minimize redundancy and collinearity within the dataset, highly correlated metrics were excluded. Subsequently, six feature importance methods based on statistical tests were employed: ANOVA, Analysis of Variance[41]; Chi2, Chi-square test[42]; LR, Logistic Regression[43]; RF, Random Forest[44]; XGBoost, eXtreme Gradient Boosting[45]; PCA, Principal Component Analysis.[46] For each method, importance scores were normalized to their respective minimum and maximum values, and features were ranked accordingly. This process identified organoid surface area and normalized mean intensity as the two most discriminative variables. To assess epithelial disruption and recovery, the 'Intestinal Organoid Recovery Score (IORS)' was calculated based on the dynamic changes in organoid surface area and normalized intensity. Fold changes in both metrics were computed relative to the onset of barrier disruption (T-0). Within the T-0 to T-7 time window, the relative AUC was calculated for both organoid surface area fold change and normalized mean intensity using a baseline normalized to 1. The recovery score was derived by summing these two AUC values and subsequently applying min-max scaling.

Immunofluorescence staining

Organoid cultures in the Gri3D® platform were fixed on day 8 using 4% paraformaldehyde (J61899, Thermo Fisher Scientific) in PBS (10010056, Thermo Fisher Scientific) for 30 min at room temperature (RT). Following three washes with PBS (5 min each), samples were permeabilized and blocked for 2 h at RT in a solution containing 1% Triton X-100 (T8787, Sigma-Aldrich) and 10% fetal bovine serum (FBS; Thermo Fisher Scientific, A5256801) in PBS. Organoids were subsequently incubated overnight at 4°C with primary stains, including DAPI (62248, Thermo Fisher Scientific), Phalloidin (ab176753, Abcam), Ki-67 (MA5-14520, Invitrogen), and E-cadherin (ab231303, Abcam), using 50 μL of staining solution per well. The cultures were then washed twice with a washing buffer

consisting of 0.3% Triton X-100 (T8787, Sigma-Aldrich) in HBSS (Hanks' Balanced Salt Solution; Thermo Fisher Scientific, 14025050), followed by incubation with appropriate secondary antibodies for 2 h at RT in the dark. After staining, samples were washed twice with washing buffer and once with PBS. Imaging was performed using a Zeiss LSM700 confocal microscope at 4×, 10×, and 20× magnifications. For each sample, 10–20 z-stacks were acquired with a step size of 20 μm.

Live/Dead assay

Organoid viability was assessed at 2 days post-treatment using the Gri3D® platform and the LIVE/DEAD™ Viability/Cytotoxicity Kit (L3224, Thermo Fisher Scientific). Prior to staining, Calcein-AM and Ethidium Homodimer-1 (EthD-1) were equilibrated at room temperature for 30 min. The culture medium was carefully aspirated via the pipetting port and replaced with 100 μL of staining solution containing Calcein-AM (1:4000) and EthD-1 (1:1000) diluted in basal medium (BM). Organoids were incubated at 37°C for 1 h. Fluorescence imaging was performed using an IN-Cell Analyzer (GE Healthcare). Image processing and quantitative analysis were carried out via DOPPL's automated fluorescence analysis pipeline, which integrates CellProfiler modules (v4.2.5) and the Tidyverse package in R (v4.3.1) for data manipulation. Fluorescence signals (Calcein-AM: ex/em ~495/515 nm; EthD-1: ex/em ~495/635 nm) were acquired across 15 Z-stacks (20 μm), followed by image deconvolution and maximum intensity projection. Organoids were automatically segmented, and the mean fluorescence intensity was quantified for each (individual) organoid.

Organoid permeability assay

Organoids cultured on the Gri3D® platform for 8 days (T-4) were incubated for 2 h at 37°C in a solution containing 0.5 mg/mL 4.4-kDa TRITC–Dextran (46944, Sigma-Aldrich) and 0.5 mg/mL 70-kDa FITC–Dextran (90718, Sigma-Aldrich). After incubation, organoids were washed three times with PBS (10010056, Thermo Fisher Scientific), followed by two washes with organoid medium, and subsequently imaged live using a Zeiss LSM700 confocal microscope.

Cytokine-induced epithelial barrier disruption assay

Following 72 h of culture on the Gri3D® platform, during which 3D organoid structures were established, the intestinal organoid culture medium was refreshed. On day 4, cytokines were introduced to the organoids to initiate Phase 1 of epithelial disruption (T-0). In this configuration, cytokines were delivered through the surrounding/basolateral medium compartment of the Gri3D® microwells, enabling controlled exposure of organoids to soluble inflammatory mediators under standardized culture conditions. Organoids were stimulated with CytoMix, a pro-inflammatory cytokine cocktail comprising TNF-α (300-01A, Thermo Fisher Scientific), IL-1β (200-01B, Thermo Fisher Scientific), and IFN-γ (300-02, Thermo Fisher Scientific), each administered at final concentrations of 5, 10, 20, or 50 ng/mL. In parallel, IFN-γ was applied as a single agent at identical concentrations. Cytokines were reconstituted at 100 μg/mL in PBS (10010056, Thermo Fisher Scientific), supplemented with 0.1% bovine serum albumin (BSA; 15260-037, Thermo Fisher Scientific). CytoMix was prepared as a 100× stock solution in organoid culture medium and directly added to the cultures without a medium change, followed by gentle homogenization. After 48 h of cytokine exposure, CytoMix was removed, and the cultures were washed by performing two to three successive medium replacements, marking the onset of the Phase 2 post-treatment recovery period (T–2).

Bioactive compound preparation and screening assay

As part of the prevention protocol, organoids cultured in the Gri3D® platform for 72 h (day 3) were supplemented with the bioactive compounds dexamethasone (DEX - 1 μM; D4902, Sigma-Aldrich), niacinamide (NAM - 0.5 mM; 0429449, DSM-Firmenich), and β-hydroxybutyrate (BHB - 1 mM; 3HB-Mg, Osaka Gas). Following cytokine withdrawal and medium washout, bioactives were reintroduced into the culture at the same concentration on day T-2 and subsequently replenished at identical concentrations on days T-4 and T-6 during routine medium changes. In the treatment protocol, the bioactive compounds – DEX at 1 μM, NAM at 0.5 mM, and BHB at 1 mM – were added to the organoid cultures on day T–2, following cytokine withdrawal and medium washout. The same concentrations were replenished on day T–4 and day T–6 during routine media replacement. DEX was initially resuspended at a concentration of 10 mM in dimethyl sulfoxide (DMSO; D2438, Sigma-Aldrich). NAM and BHB were resuspended at concentrations of 500 mM and 250 mM, respectively, in phosphate-buffered saline (PBS; 10010056, Thermo Fisher Scientific). Stock solutions of each bioactive compound were subsequently diluted in the cell culture medium to achieve their respective final working concentrations.

RNA extraction

Total RNA was extracted from human intestinal organoid samples using the Agencourt RNAdvance Tissue Kit (A32649, Beckman Coulter), following the manufacturer's protocol. Tissue disruption and lysis were carried out using the FastPrep-24™ instrument (MP Biomedicals) at a speed setting of 6.0 for two cycles of 1 min each. A total of 400 μL of lysate was processed per sample, and RNA was eluted in 50 μL of nuclease-free water. RNA concentration was quantified using the Quant-iT™ RiboGreen RNA Assay Kit (R11490, Invitrogen) on a SpectraMax® F1 Microplate Reader (Molecular Devices). Integrity was assessed with the Fragment Analyzer 96-capillary system (Agilent Technologies) using the Standard Sensitivity RNA Analysis Kit (DNF-471-0500). We consistently obtained high-quality RNA from all organoid samples, with an average yield of 0.36 μg per sample. Notably, all samples

CellPress
OPEN ACCESS

achieved an RNA integrity number (RIN) value of 10, indicating excellent RNA integrity. These RNA samples were subsequently used for Illumina library preparation and high-throughput sequencing.

Transcriptome profiling: Bulk RNA sequencing (RNA-seq)

A total of 300 ng of RNA per sample was used to generate sequencing libraries with the Illumina Stranded mRNA Library Prep Kit, following the manufacturer's protocol. The workflow includes a bead-based mRNA capture, followed by fragmentation, first- and second-strand synthesis, end repair, 3′ adenylation, and indexed linker ligation steps. The adapter-ligated cDNA library fragments were next enriched by PCR using 13 cycles of amplification. From the extracted total RNA, 90 individual barcoded stranded mRNA-seq libraries were successfully prepared with an average yield of 35.4 ng/μL. Two equimolar pools of 78 and 12 unique dual (UD) indexed libraries were sequenced paired-end (PE) with 59 bases per read on four individual P3 100 NextSeq 2000 flow cells. The first dark cycle was introduced to the sequencing run to improve the flow-cell cluster detection. 5.0 billion PE reads passing the chastity filter (>Q30) were generated, with an average of 46.2 million reads per sample, with a standard deviation of 11.8 million reads (26%) using the Illumina NextSeq 2000 platform.

RNA-seq data analysis

Sequencing reads were mapped to the human hg19 genome using HISAT2[90] with the following parameters: hisat2 -k 5 –seed 42 –rna-strandness RF. Gene-level counts were generated using featureCounts.[91] Only uniquely mapped reads were used for counting genes. To account for differences in sequencing depth, normalization was performed using the trimmed mean of M-values (TMM) method, as implemented in the limma package (Bioconductor).[92] Data analysis was conducted with R version 4.2.1. Differential gene expression analysis was carried out using variance modeling at the observational level (voom)[96] as implemented in the limma package. A gene was considered differentially expressed if the fold change between groups exceeded 2 and the p-value was less than 0.05. The significance of differential expression was tested using a moderated t test, as implemented in the limma package of R. P-values were adjusted for multiple testing using the Benjamini-Hochberg procedure.[97] Gene set enrichment analysis (GSEA) was conducted using the ClusterProfiler package in R. The analysis employed a ranked list of genes and gene set collections obtained from the Molecular Signatures Database (MSigDB). The GSEA was performed with the following parameters: 10,000 permutations, a minimum gene set size of 10, and a maximum gene set size of 800.

Microarray data from patient samples via the Gene Expression Omnibus (GEO: GSE52746)

Normalized gene expression values were obtained directly from the GEO repository. Probe identifiers from the corresponding microarray platform were mapped to Ensembl gene identifiers to enable cross-platform comparisons. Differential expression was assessed by calculating fold changes, which were then compared to those derived from RNA-seq analysis for genes common to both datasets. All computational analyses were conducted using R (version 4.2.1).

Figure design and schematics

All schematic illustrations and figure designs presented in this manuscript were created using CorelDRAW Graphics Suite X8 (version 18.0.0.448, Corel Corporation) or BioRender.com.

QUANTIFICATION AND STATISTICAL ANALYSIS

Organoid morphology, cytotoxicity, and permeability were quantified using automated image-analysis pipelines as described above. Outputs were plotted and statistically analyzed using GraphPad Prism v9.3.0, and two-way ANOVA with Bonferroni's post-test was performed where appropriate ($p < 0.05$ (*), $p < 0.01$ (**), $p < 0.001$ (***), and $p < 0.0001$ (****)). As indicated in the captions of each representative figure in the main manuscript and Supplementary Information, all data are presented as mean ± standard deviation (SD) or with 95% confidence intervals (CI), as appropriate. For all experimental conditions assessing organoid morphology and intervention effects, the number of organoids analyzed is indicated in the captions of each representative figure in both the main manuscript and the supplemental information. Transcriptomic data were analyzed using R (version 4.2.1), with p-values adjusted for multiple comparisons via the Benjamini–Hochberg procedure[97] to control the false discovery rate (FDR).

Cell Reports Methods, Volume *6*

Supplemental information

A scalable microarrayed organoid platform for precision modeling of intestinal epithelial damage and recovery

Marine Meyer, Aline Roch, Juliane Dervaux, Laurent Ferrier, Evarist Planet, Julien Duc, Camilla Ceroni, Mariana Costa, Brice Chambrin, Jens Stolte, Sylviane Métairon, Abhilash Kannan, Sylke Hoehnel-Ka, Marine R.-C. Kraus, Patrick Descombes, Nathalie Brandenberg, Mario Noti, and Omid Mashinchian

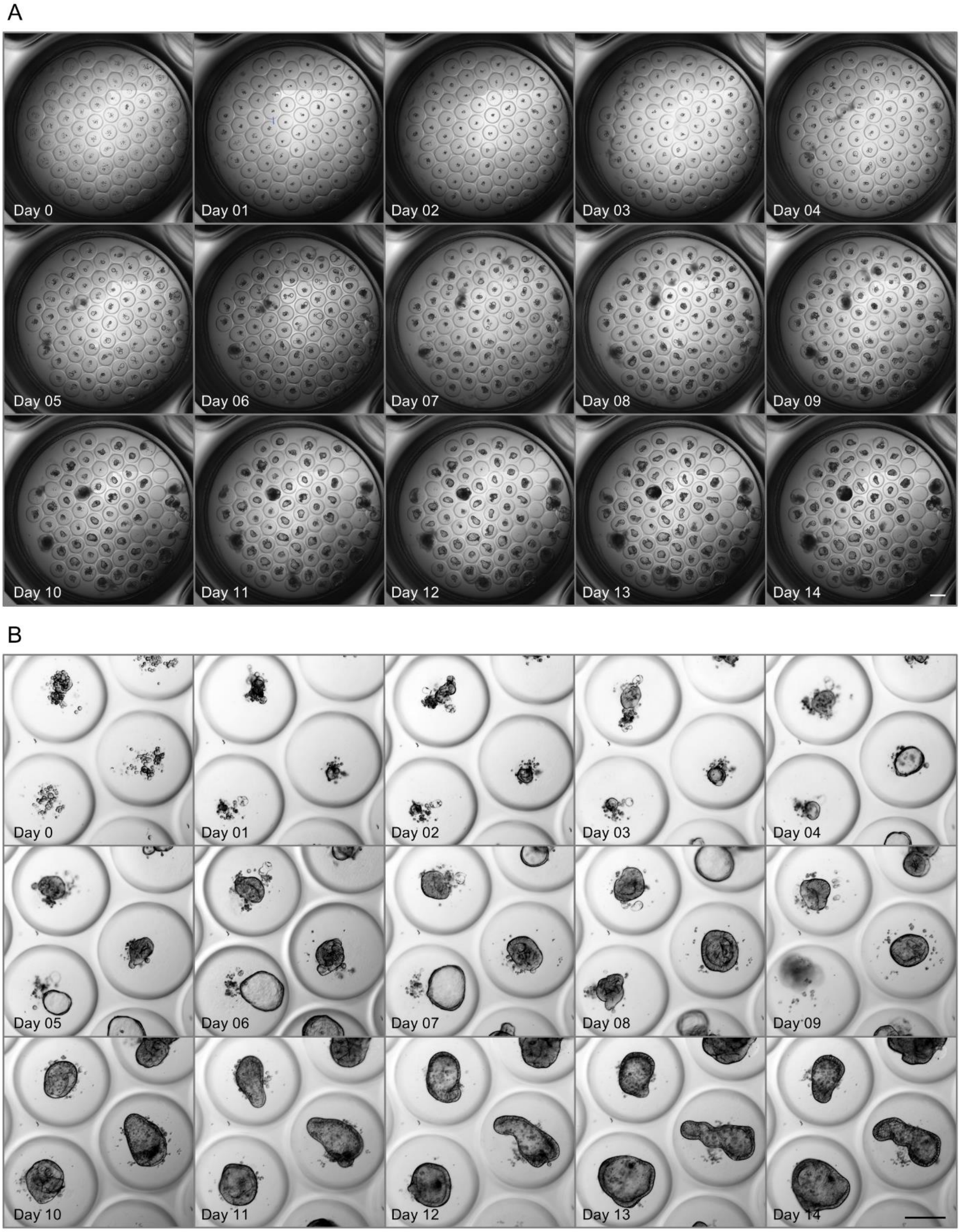

Figure S1. Time-Lapse Microscopy of Intestinal Organoid Growth and Development over 14 Days, related to Figure 1. A. Representative low-magnification brightfield images capturing the overall growth and expansion pattern of multiple organoids cultured within microwells, tracked daily from Day 0 to Day 14. The scale bar denotes consistent magnification across all images: 500 μm. **B.** High-magnification brightfield images illustrating morphological changes and detailed structural development of individual intestinal organoids within microwells over the same 14-day period, highlighting the transition from initial small cellular clusters to mature, differentiated organoid structures exhibiting distinct epithelial morphologies and lumen formation. The scale bar indicates uniform magnification for all high-magnification panels: 300 μm.

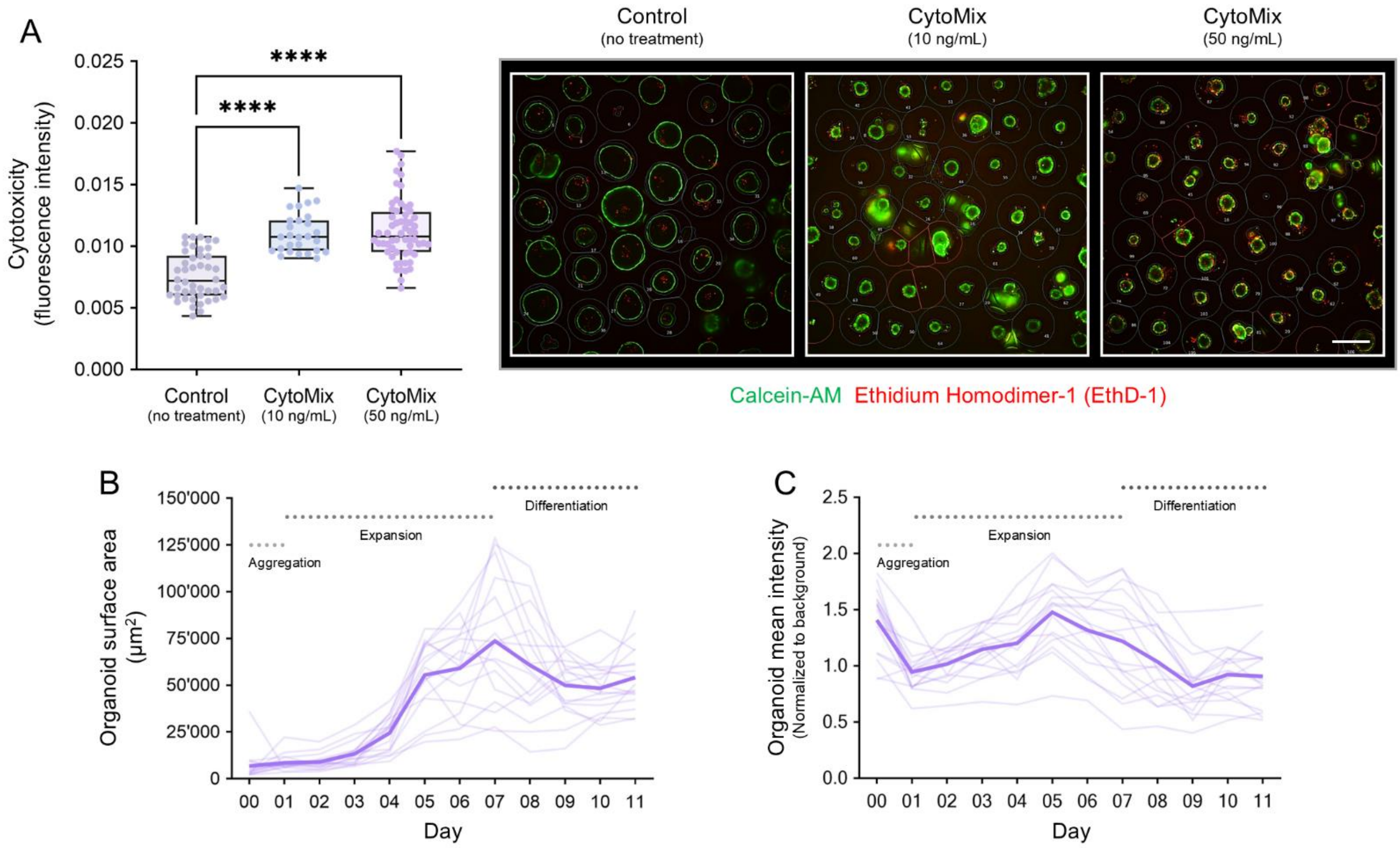

Figure S2. Cytotoxicity and Morphodynamic Assessment of Intestinal Organoids During Development and Cytokine-Induced Stress, related to Figures 1 and 2. A. Cytotoxicity was assessed by measuring the fluorescence intensity of Ethidium Homodimer-1 (EthD-1) in control (untreated), CytoMix-treated (10 ng/mL), and high-dose CytoMix-treated (50 ng/mL) organoids after 48 hours. Quantitative data are presented as box plots, with statistical significance indicated by asterisks (n ≥ 27 organoids). Each dot represents an individual organoid. Two-way ANOVA with Bonferroni's post-test was performed using GraphPad Prism (****p < 0.0001). Representative fluorescence microscopy images depict viable cells (green; Calcein-AM) and compromised or dead cells (red; Ethidium Homodimer-1 (EthD-1)) under each treatment condition. Images are shown as maximum intensity projections across 15 z-planes, with a step size of 20 µm. Scale bars represent 500 µm and indicate uniform magnification across all images. **B.** Quantitative analysis of organoid surface area dynamics during aggregation, expansion, and differentiation phases. Individual organoid trajectories are shown in light purple, and the mean trajectory is highlighted in dark purple (mean, n ≥ 17 organoids). **C.** Corresponding organoid mean intensity measurements normalized to background intensity, reflecting changes in organoid density and structural complexity during culture progression (mean, n ≥ 17 organoids).

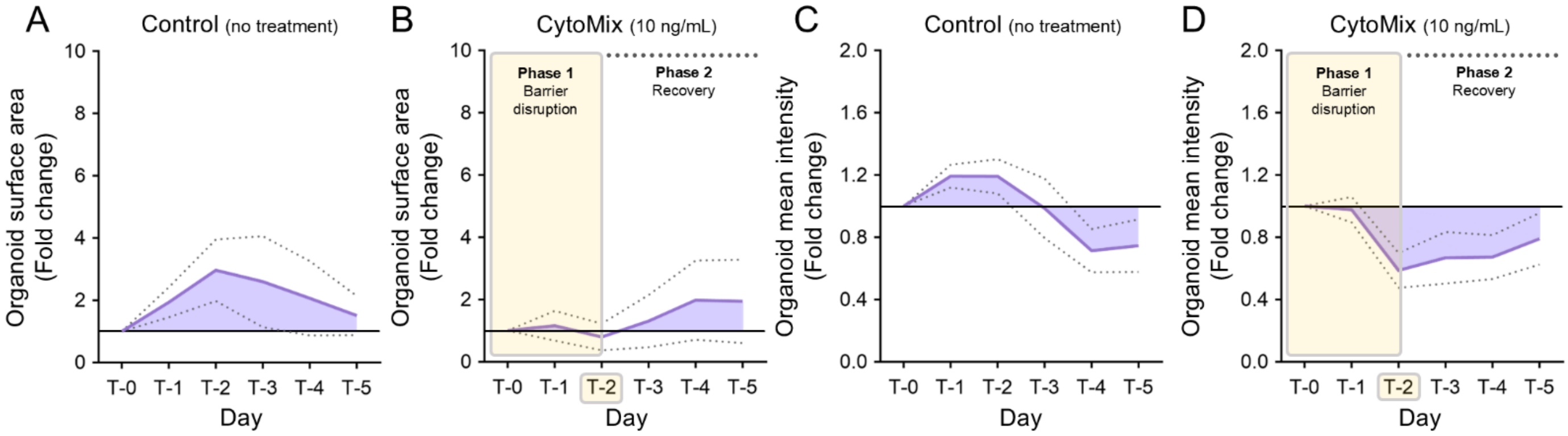

Figure S3. Longitudinal feature dynamics underlying IORS calculation in untreated and CytoMix-challenged intestinal organoids, related to Figure 3. A–B. Time-course analysis of organoid surface area, expressed as fold change relative to baseline, in untreated control organoids and organoids exposed to CytoMix (10 ng/mL). **C–D**. Time-course analysis of normalized organoid mean intensity, expressed as fold change relative to baseline, in untreated control organoids and CytoMix-treated organoids. The shaded region indicates Phase 1, corresponding to cytokine-induced epithelial barrier disruption, while the subsequent period represents Phase 2, corresponding to post-cytokine recovery. Solid lines represent mean values, shaded purple bands indicate variability around the mean, and dotted lines indicate the spread of individual organoid trajectories. These longitudinal feature dynamics provide the underlying input parameters used for calculating the Intestinal Organoid Recovery Score (IORS) and illustrate the reversible epithelial injury phenotype induced by CytoMix 10 ng/mL.

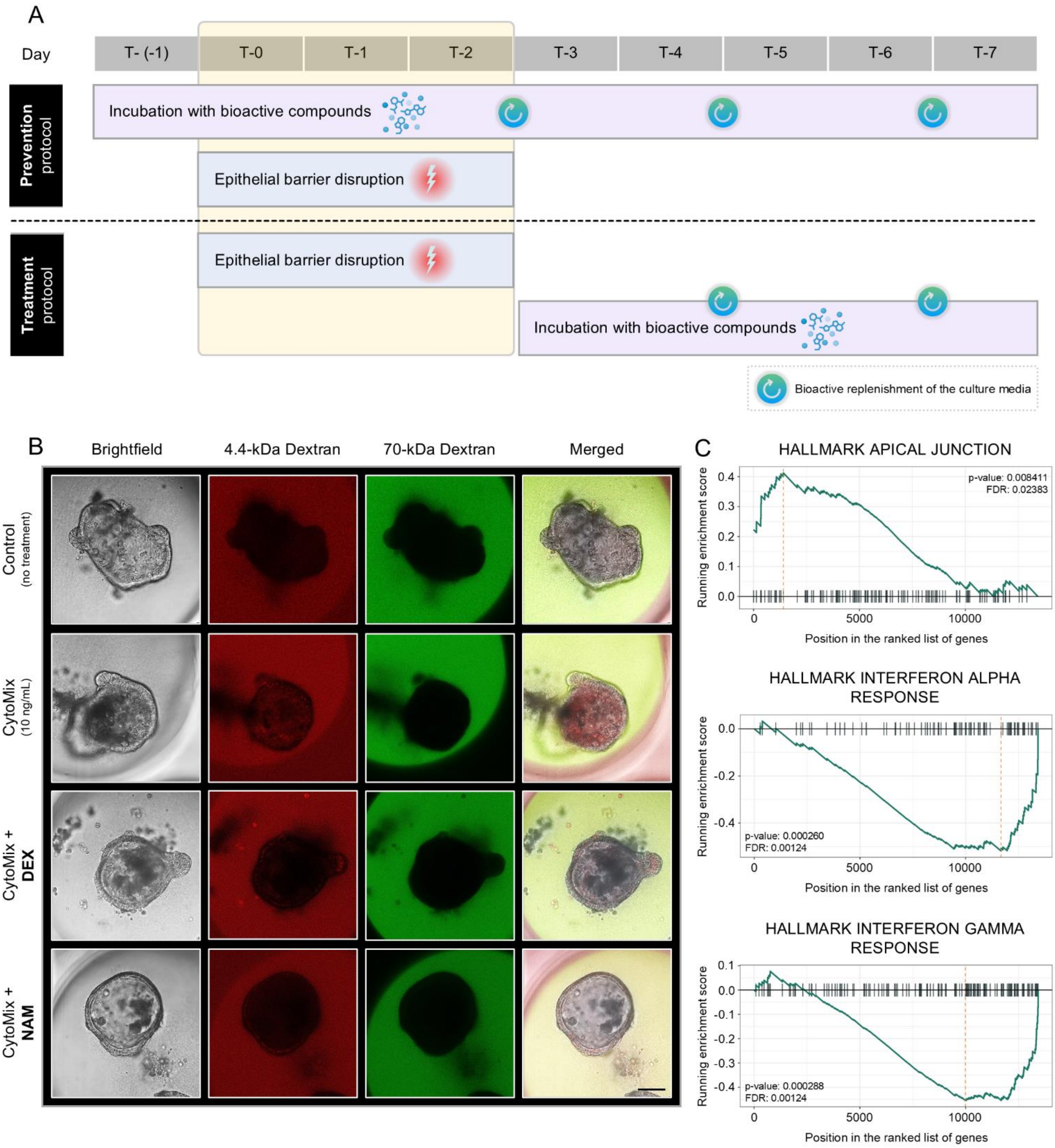

Figure S4. Preventive and Therapeutic Effects of Dexamethasone (DEX) and Nicotinamide (NAM) on Cytokine-Induced Barrier Disruption Assessed by Permeability Assays and Transcriptomic Profiling, related to Figures 4 and 5. A. Schematic outline of experimental timelines and treatment protocols: Prevention (Therapeutic agents administered prior to cytokine exposure) and treatment (interventions initiated post-epithelial disruption) protocols, specifying the timing of cytokine exposure, recovery phases, and administration of bioactive compounds. **B.** Representative microscopy images showcasing barrier integrity using fluorescently labeled dextran permeability assays (4.4-kDa and 70-kDa dextrans) under various experimental conditions: Control (no treatment), CytoMix (10 ng/mL) alone, CytoMix plus dexamethasone (DEX), and CytoMix plus nicotinamide (NAM). Scale bars reflect uniform magnification across all images: 50 µm. **C.** Gene set enrichment analysis (GSEA) plots demonstrate significantly altered hallmark gene signatures related to epithelial barrier function (apical junction integrity) and inflammatory signaling pathways (Interferon Alpha and Interferon Gamma Responses), highlighting significant modulation following DEX treatment. Enrichment scores, nominal *p*-values, and False Discovery Rates (FDR) values are provided.
